# Effects of hydro-meteorological and geological disasters on vaccine-preventable disease outbreaks and routine immunisation amongst children: A scoping review

**DOI:** 10.1371/journal.pgph.0005712

**Published:** 2026-06-12

**Authors:** Isabel T. Barnidge, Andrea J. F. Ferreira, Sari Kovats, Sofia R. M. Velasco, Alastair Leyland, Luiz Augusto Galvao, Gervasio F. Santos, Enny S. Paixao, Mauricio L. Barreto, Elizabeth B. Brickley, Julia M. Pescarini, on behalf of the Global Health Research Unit on the Social and Environmental Determinants of Health Inequalities (SEDHI)

**Affiliations:** 1 Faculty of Epidemiology and Population Health, London School of Hygiene & Tropical Medicine, London, United Kingdom; 2 Centre for Data and Knowledge Integration for Health (CIDACS), Gonçalo Moniz Institute, Oswaldo Cruz Foundation, Salvador, Bahia, Brazil; 3 Faculty of Public Health and Policy, London School of Hygiene & Tropical Medicine, London, United Kingdom; 4 MRC/CSO Social & Public Health Sciences Unit, University of Glasgow, Glasgow, United Kingdom; 5 Centre for Global Health, Oswaldo Cruz Foundation, Rio de Janeiro, Rio de Janeiro, Brazil; 6 Faculdade de Economía, Universidade Federal da Bahia, Salvador, Brazil; Fatima Jinnah Women University, PAKISTAN

## Abstract

Hydro-meteorological and geological disasters can pose serious harm to community health, such as an increased risk of infectious diseases and related mortality. We conducted a scoping review to compile the available literature on the effects of hydro-meteorological and geological disasters on routine immunisation and vaccine-preventable disease (VPD) outbreaks among children and adolescents under 18 years. We searched Medline, Embase, Global Health, Scopus, and Web of Science for original studies and systematic reviews on the topic published by the 21st of October 2024. We included studies that quantitatively analysed the effect of any of the thirty Emergency Events Database (EM-DAT) recognised hydro-meteorological and geological disasters (or those fitting EM-DAT criteria) on a comprehensive list of World Health Organization (WHO)-recommended vaccines and the 23 diseases against which they prevent. We included 26 studies, of which 19 concerned with vaccine-preventable disease outbreaks and seven focused on routine immunisation disruption.. Our review indicates that floods, cyclones, droughts, extreme temperatures, tsunamis, and earthquakes may result in vaccine-preventable disease outbreaks and routine immunisation disruption, and identified young children and refugees as at-risk populations. Flooding was reported to be associated with water-borne and vector-borne vaccine-preventable disease outbreaks such as malaria. Poor water, sanitation and hygiene (WASH) were further recognised as facilitating conditions for vaccine-preventable disease outbreaks after disasters. The vaccination studies reported reduction in routine immunisation rates in the months following a disaster, considering infrastructural damage to health facilities and vaccine storage issues as common causes. Finally, there was significant knowledge gap on the effects of specific disasters (e.g., wildfires and volcanic eruptions) and diseases (e.g., influenza, yellow fever, malaria, and dengue).In conclusion, our review reinforce the need for better policies to ensure vaccine equity, resilient health system and safe WASH to prevent vaccine-preventable disease (VPD) outbreaks following hydro-meteorological and geological disasters.

## Introduction

Disasters occur when hazardous events coincide with conditions leading to exposure and vulnerability [1]. The Emergency Events Database (EM-DAT) describes a disaster as a natural (geophysical, hydrological, meteorological, climatological, biological or extraterrestrial [2]) or technological hazard resulting in at least ten deaths, affecting one hundred people, eliciting a declaration of a state of emergency, or bringing about an international call for assistance [3]. Since 1980, hydro-meteorological and geological disasters, such as earthquakes, floods, and droughts, have affected 7.1 billion individuals and prompted over 1.5 million deaths [3]. In the last 50 years, disasters have increased fivefold, rising from 711 in 1970–1979–3,536 in 2000–2009 [4]. Aside from improvements in recording, this escalation is thought to be the result of factors such as shifts in human settlement patterns and climate change [5]. Without substantial modifications, disasters are projected to reach 560 annually (from 400 in 2015) by 2030 [6], posing significant risk to human health and lives.

Much of global disaster coverage focuses on blunt trauma-induced deaths and injuries; however, these events also frequently introduce opportunities for infectious disease spread. This is especially true when a disaster affects a population with poor pre-existing water, sanitation & hygiene (WASH) infrastructure, nutrition and/or vaccination status, and healthcare accessibility [7]. In their literature review, Walika et al. found 108 disaster events associated with infectious disease outbreaks between 2000 and 2020 [8]. Nevertheless, disaster risk reduction generally overlooks disease prevention and control [9]. Gavi, the Vaccine Alliance has recently positioned vaccination as a climate adaptation strategy. The organisation argues that vaccinating populations against climate-sensitive diseases mitigates the disruption and strain climate change places on health systems [10].

Whilst disasters may make an unvaccinated or under-vaccinated population immediately vulnerable to disease spread, they may also disrupt routine immunisation schedules, putting a population at risk of an eventual vaccine-preventable disease (VPD) outbreak [7]. The continuation of vaccination services amidst disaster speaks to a healthcare system’s resilience, or its ability to plan for, carry out core functions during, and successfully adapt activities to a crisis [8] Maintaining high community vaccination coverage prior to and ensuring emergency vaccine distribution in the wake of a disaster is critical to preventing outbreaks and protecting population health [9]. The interruption or delay in the delivery of routine vaccines especially affects children, putting this age group in particular danger of acquiring diseases against which they are normally protected [10].

Given the increasing risk of hydro-meteorological and geological disasters, and the urgent need for climate adaptation, particularly in regions that already bear a disproportionate burden of infectious diseases, it is imperative to understand how these disasters affect childhood vaccination and the occurrence of VPD outbreaks to better protect children’s health.

We conducted a scoping review aimed at compiling the literature available concerning the effect of hydro-meteorological and geological disasters on routine immunisation and VPD outbreaks amongst children, or those under eighteen. This critical narrative review was structured to address five objectives: the first two aimed to describe the current literature concerning disaster-related disruption of (i) childhood immunisation programmes and (ii) VPD outbreaks by regions of study, publication years, disasters in question, vaccine types, and diseases covered. The other three aimed to (iii) investigate the outbreaks by social and demographic factors, (iv) examine the impact of floods on WASH-related and vector-borne VPDs, and (v) identify and critically evaluate common disaster-induced obstacles to routine childhood immunisation in the aftermath of a hydro-meteorological and geological disaster.

## Materials and methods

### Study design

We conducted this scoping review in accordance with the guidelines of the 2018 Preferred Reporting Items for Systematic reviews and Meta-Analyses extension for Scoping Reviews (PRISMA-ScR) (See S1 Prisma Checklist) [11]. We gathered, presented, and critically evaluated the research published regarding disaster-associated childhood immunisation disruption and VPD outbreaks.

### Definitions

Comprehensive definitions of the population, intervention/exposure, comparison and outcomes (PICO) components of this study can be found in Table 1. In summary, we looked at the effect of exposure to a simplified classification of EM-DAT recognised and Integrated Research on Disaster Risk (IRDR)-classified disaster events or perils on the coverage and uptake of WHO recommended childhood vaccines or the cases, deaths, and measures of association for the 23 corresponding VPDs amongst UNCRC-defined children globally (See Table 1 for PICO strategy and S1 Fig for EM-DAT disaster definition).

**Table 1:** Population, Intervention/Exposure, Comparison, and Outcomes (PICO) definitions and their inclusion/exclusion criteria.

PICO Component	Definition	Inclusion Criteria	Exclusion Criteria
Population	Children, defined according to the United Nations Convention on the Rights of the Child (UNCRC) as individuals under 18 or the legal adulthood age of their country.	Studies looking at children under 18 years.	Studies that did not provide specific measures of association for children (i.e., grouping with adults).
Intervention/Exposure	A disaster was defined by EM-DAT criteria: a geophysical, hydrological, meteorological, or climatological hazard, as understood by the Integrated Research on Disaster Risk (2014 IRDR), that caused at least ten deaths, affected one hundred people, elicited a declaration of a state of emergency, or brought about an international call for assistance [12].	Studies focusing on the effects of one or more events that qualify as a disaster (See S2 Table).	Studies focusing on biological and extraterrestrial events, which are IRDR-recognised natural hazards but irrelevant to this paper.
Comparison	Studies comparing outcomes of children exposed or unexposed to a disaster. Multiple comparison groups were considered, depending on study design, but included: (i)residing in an area affected versus unaffected by disaster(s) (ii)residing in areas pre- versus post-disaster(s) (iii)outbreak scenarios post-disaster(s), considering prior absence of disease. For an area to be considered unaffected, the study authors had to identify it as such.	Studies presenting children’s outcomes in areas affected versus unaffected by disaster, pre- versus post- disaster, or only post-disaster in the case of outbreak scenarios.	Studies not explicitly presenting children’s outcomes in areas affected versus unaffected by disaster, pre- versus post- disaster, or only post-disaster in the case of outbreak scenarios.
Outcomes	Outcomes included: (i)Coverage and uptake of vaccines, as recommended by WHO routine childhood immunisation. (ii)Cases, deaths, and measures of association pertaining to diseases preventable by WHO recommended immunisations.	All WHO-recommended childhood vaccines and the 23 diseases against which they provide protection are included in this review.^1^	Cases of disease without a primary identified pathogen included in our outcome list.

1 As of April 2014, the WHO recommends that children receive the following vaccines: *Haemophilus influenzae* type B (Hib), polio, Bacillus Calmette-Guérin (BCG), hepatitis B, pneumococcal, rotavirus, measles, rubella, human papillomavirus (HPV), DTP-containing [13,14]. Please note that BCG is recommended universally only for high-burden settings for Tuberculosis and/or leprosy. The organisation further proposes numerous vaccines for (i) children living in particular regions: Japanese encephalitis (JE), yellow fever, and tick-borne encephalitis, (ii) belonging to specific populations: typhoid, cholera, meningococcal, hepatitis A, rabies, dengue* and malaria**, and (iii) acquiring immunisation through certain programmes: mumps, seasonal influenza, and varicella [13].

* Dengue and malaria** were included in analysis; however, these vaccinations were only recently added to the WHO list of recommended routine immunisations and were not expected to be covered in abundance in the literature.

The events included met EM-DAT criteria but were not necessarily registered in the database. The magnitude of the events was not considered, meaning that events occurring over long periods of time, analysed as a series, or without the number of affected people were included (See S1 Fig and S2 Table).

### Data sources and search

A search was conducted on the 21st of October 2024 on five bibliographic databases: Medline, Embase, Global Health, Scopus, and Web of Science looking at papers analysing the effect of disasters on routine immunisation and VPD outbreaks amongst children. The initial search was carried out on Medline, and terms were adapted to other databases as necessary (See S1 Table for the Medline search strategy). Search terms fell under four concepts: vaccines (names for WHO-recommended vaccines), vaccine-preventable diseases (the diseases covered by WHO-recommended vaccines), disasters (those satisfying EM-DAT criteria), and children (synonyms for different child age groups).

Inclusion and exclusion criteria were defined prior to a thorough review of the literature (See Table 1). We have included (i) original and analytical studies, emerging from any country or region of the world, and having been published at any point in time, (ii) studies containing a comparison group (i.e., children pre-disaster or residing in an area unaffected by a disaster) or (iii) descriptive studies reporting on VPD outbreaks.

We excluded studies that grouped diseases (i.e., gastroenteritis, diarrhoea, and respiratory infections) when cases were not attributed to vaccine-preventable pathogens. We further excluded studies written in any language apart from those spoken by the reviewers (English, Spanish, and Portuguese). The search terms were entered in English. Studies were screened by title and abstract in duplicates (IB and SMV) using Rayyan, with discrepancies being agreed upon with a third reviewer (JMP).

### Data extraction and analysis

After full-text screening, a single reviewer (IB) extracted the following data in a pre-defined extraction form containing: author, year published, title, study aims, study design, study setting, type of disaster, date(s) of disaster, vaccine or VPD of interest, populations affected and unaffected, outcome for affected and unaffected populations, main results and conclusions.

Following data extraction, the findings were summarised in tables by IRDR-classified disaster type (See S1 Fig) and in a narrative synthesis, including results and interpretation of the findings as stated by the authors. Given the heterogeneity of disaster types, geographic contexts, vaccines, and disease outcomes across studies, this structuring organizes the findings to best highlight key relationships and themes. The narrative synthesis followed JBI guidelines and was organised to match study objectives, which included two topics on the overall effects of disaster-related disruption on (i) childhood immunisation programmes and on (ii) VPD outbreaks by regions of study, publication years, disasters in question, vaccine types, and diseases covered; and three topics aimed to go in depth on the effects of social and demographic factors, key disaster/health events relationships (i.e., impact of floods on WASH-related and vector-borne VPDs), and common disaster-induced obstacles to routine childhood immunisation.

## Results

### Summary of studies

**Fig 1:**
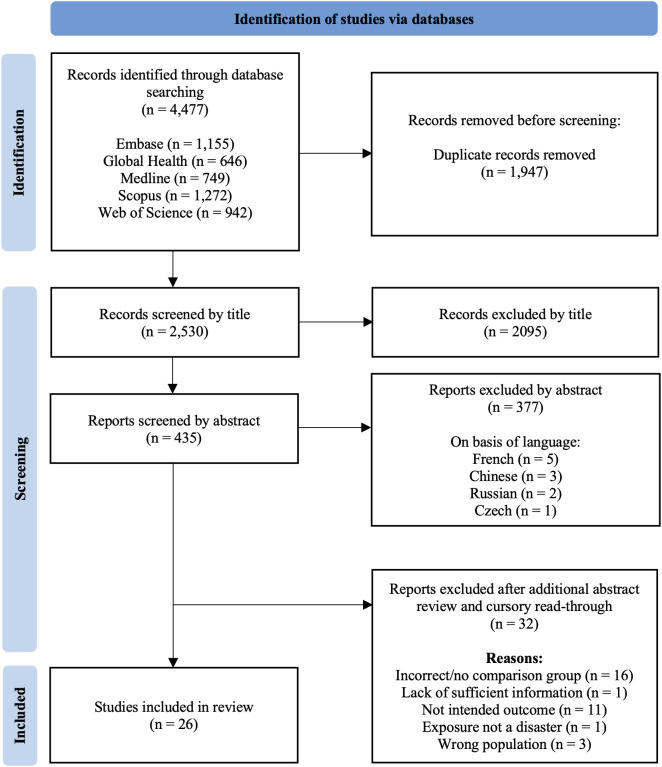
PRISMA 2020 flow diagram for scoping review.

Our search yielded 4,477 records. After removing duplicates and applying inclusion and exclusion criteria, we extracted information from 26 studies (See Fig 1). The studies selected were all published between 1991 and 2024 (See Tables 2 and Table 3). 12 studies were published between 2010–2019, studies from the 1990s and 2000s were rather sparse.

**Table 2:** Summary of Included Studies on Disaster-Related Vaccine-Preventable Diseases by Family of Event, Ordered by Year of Publication.

Author & Year		Study Design & Period	Population, Sample & Comparison	Setting & Region	Identified Disaster & Year	Outcome	Analysis & Methods	Results	Conclusions
GEOPHYSICAL									
**Mohan et al.** **(2006)** [15]		Descriptive: Outbreak investigation December 2004 to January 2025	An estimated 18,952 children ≤14 residing in tsunami-affected (N = 14,284) and unaffected (N = 4,668) villages of Cuddalore	Tamil Nadu, India (South Asia)	2004 Indian Ocean earthquake and tsunami (December 26, 2004)	Measles cases and incidence (per 1000), by age group	Cases defined by WHO guidelines and recorded by district health care facilities.	Cases *Tsunami-affected* **0-4 yrs:** 31 (7.1/1000) **5-9 yrs:** 32 (6.7/1000) **10-14 yrs:** 8 (1.6/1000) *Tsunami-unaffected* **0-4 yrs:** 11 (7.7/1000) **5-9 yrs:** 16 (10.2/1000) **10-14 yrs:** 2 (1.2/1000)	With similar incidence of measles amongst children in tsunami-affected and unaffected villages, the study concluded that the measles outbreak in the Cuddalore district occurred independently of the tsunami.
**Karmakar et al.** **(2008)** [16]		Descriptive: Outbreak investigation October 14 to December 17, 2005	All children <5 in the Tangdar (N = ~8,840) No comparison (outbreak)	Kashmir, India (South Asia)	2005 Kashmir earthquake (October 8, 2005)	Acute diarrhoeal disease (ADD) and weekly attack rates	Cases registered with the Tangdar Community Health Centre (CHC) Hospital or Tangdar-based primary health centres and rehabilitation camps. 12 random stool and rectal swab samples of ADD patients at Tangdar CHC Hospital tested for source of outbreak.	Cases **Total:** 1,633 The week of October 22 saw the greatest number of ADD cases (N=281). Attack Rate The weekly attack rate was as low as 1.48% and as high as 3.17%. 20% overall attack rate. Sample Results 3 out of 12 samples returned positive for rotavirus.	Rotavirus was the most common pathogen amongst ADD cases sampled. The virus was able to spread in part due to overcrowding and poor WASH conditions.
**Balasubramaniam & Roy** **(2012)** [17]		Descriptive: Outbreak investigation December 29, 2004 to January 3, 2005	Children aged 3 to 8 in tsunami-affected and unaffected villages of Cuddalore	Tamil Nadu, India (South Asia)	2004 Indian Ocean earthquake and tsunami (December 26, 2004)	Measles cases	Cases registered with the office of the Deputy Director of Public Health (DDPH). Serological testing was completed on the first three cases.	Cases **Total:** 27 **Tsunami-affected:** 14 **Tsunami-unaffected:** 13 All the measles cases from the tsunami-unaffected area of Cuddalore came from the same street of a single village (Poovalai).	The study found the measles outbreak to be independent of the tsunami. Vaccination did not successfully prevent nor entirely control the outbreak. That said, it is questioned whether the vaccine was properly stored or distributed.
**Zhang et al.** **(2013)** [18]		Descriptive: Surveillance 2005 to 2011	Children <9 in Longnan City pre- and post-disaster	Gansu Province, China (East Asia & the Pacific)	2008 Wenchuan earthquake (May 12, 2008)	Japanese encephalitis (JE) & malaria cases	Cases of JE & malaria pre- and post-earthquake, reported by the Control Disease Center of Longnan City.	JE Cases **Pre-disaster:** 17 (2005), 32 (2006), 18 (2007) **Disaster year:** 9 (2008) **Post-disaster:** 5 (2009), 3 (2010), 5 (2011) Malaria Cases Only one case of malaria recorded in a child under 9.	The study concluded that the Wenchuan earthquake did not trigger a significant change in the number of cases of Japanese encephalitis or malaria.
**Barzilay et al.** **(2024)** [19]		Descriptive: Outbreak investigation October 20, 2010 to October 20, 2012	Children <5 in all ten Haitian administrative departments No comparison (outbreak)	Haiti (Latin America & the Caribbean)	2010 Haiti earthquake (January 12, 2010)	Cholera cases, hospitalisations, and fatalities	Cases had to meet an adjusted version (inclusive of those <5) of the WHO cholera definition. Data reflects that published by Haiti’s Ministry of Public Health and Population. Stool was randomly sampled from all departments for *V.cholerae* diagnostic testing.	*Percentage of total number of cholera cases, hospitalisations, and fatalities amongst children <5 years in parentheses.* Cases 78,938 (13.1%) Hospitalisations 34,394 (43.6%) Fatalities 583 (7.8%)	It is difficult to monitor cholera incidence in young children, as they commonly suffer from diarrhoea. That said, in the first six months of Haiti’s cholera epidemic, those 2 to 5 were the cohort most seropositive for cholera. The authors argue that young children (<5) should not be immediately disregarded in cholera outbreak investigations.
HYDROLOGICAL									
**Uddin Ahmed et al.** **(1991)** [20]		Descriptive: Medical record review Pre-flood period: January to July 1988 Flood period: August 1988 to January 1989	Children ≤12 hospitalised at Mymensingh Medical College before and during monsoon-induced flooding	Mymensingh, Bangladesh (South Asia)	Floods brought on by 1988 monsoon	Rotavirus hospitalisations	Hospitalised cases of rotavirus-positive diarrhoea pre- and during monsoon flooding by age group	Cases *Pre-flood period* **0-5 mo:** 2 | **6-11 mo:** 6 **1 yr:** 9 | **2 yr:** 3 **3-5 yr:** 2 | **6-12 yr:** 1 *Flood period* **0-5 mo:** 1 | **6-11 mo:** 13 **1 yr:** 14 | **2 yr:** 9 **3-5 yr:** 21 | **6-12 yr:** 22	The uptick in rotavirus cases between August 1988 and January 1989 was likely due to flood-related contamination of food and drinking water. People also suffered from poor nutrition during this time, making their bodies more susceptible to infection.
**Van Middlekoop et al.** **(1992)** [21]		Descriptive: Surveillance December 1, 1987 to November 14, 1988	Children <5 in the Natal/KwaZulu province No comparison (outbreak)	Natal/KwaZulu, South Africa (Sub-Saharan Africa)	1987 South Africa floods (September 25 to 29)	Poliomyelitis cases and fatality rate	Cases of poliomyelitis, as captured by surveillance system. Hospitals in the region were tasked with reporting cases. A little over half (51%) of the cases were confirmed virologically.	Cases 304 in children <5 412 amongst all age groups 405 (98.3%) of these patients were Black. 46 (11.2%) of these patients had been fully immunised against the disease. Case Fatality Rate 6.9% in children <5 8% amongst all age groups	No one factor was determined to cause the outbreak. The floods in September, however, disrupted vaccination services and polluted water sources, making way for wild poliovirus. A vaccination campaign was enacted in response to the outbreak.
**Schwartz et al.** **(2006)** [22]		Analytical: Cross-sectional 1987 to 2004	Patients <15 at the International Center for Diarrheal Disease Research, Bangladesh (ICDDR, B) during flood vs non-flood periods	Dhaka, Bangladesh (South Asia)	Four flood periods: 1. Sep 6 to Oct 13, 1988 2. Jul 25 to Oct 13, 1998 3. Jul 20 to Aug 21, 2004 4. Sep 16 to Oct 24, 2004	Odds ratios (OR) and diarrhoea cases during flood versus non-flood periods, by age group. Proportion of daily diarrhoea cases attributed to *V. cholerae* and rotavirus.	Proportion differences determined by Pearson’s X^2^ test. Patient stool samples randomly selected for microorganism screening.	Cases, OR (95% CI) *Flood vs. non-flood periods* **Under 2:** 690 vs. 695 OR 0.59 (0.52, 0.68), *p* <0.001 **2 to 4 yrs:** 246 vs. 133 OR 1.32 (1.06, 1.66), *p* = 0.002 **5 to 14 yrs:** 238 vs. 137 OR 1.32 (1.05, 1.65), *p* = 0.01 Proportion *Flood vs. non-flood periods* ***V. cholerae*:** 37% vs. 20% *p* < 0.001 **Rotavirus:** 17% vs. 26% *p* = 0.002	The authors identified the pathogen *V. cholerae* as the likely source of the diarrheal epidemics tied to four floods between 1988 and 2004. Flood-related diarrheal epidemics were linked to a smaller extent to additional pathogens commonly transmitted via faecal-oral pathway (i.e. *Shigella*, *Salmonella*, etc).
**Ding et al.** **(2014)** [23]		Mixed method: Descriptive analysis, case-crossover, stratified Cox models & years lived with disability (YLD) calculation January 2005 to December 2010	Children ≤14 in Mengcheng County during flood/waterlogging* vs non-flood/waterlogging periods *Waterlogging refers to submergence or moisture/wet damage resulting from long-lasting steady precipitation.	Anhui Province, China (East Asia & the Pacific)	2007 Huaihe River floods (May to October 2007) 1. Severe flooding: Jul 3-9 2. Continuous rainfall: Jul 15-26 3. Waterlogging: Jul 26-Aug 7	Malaria cases and & epidemiological burden attributed to flooding/waterlogging	Data was obtained from the National Notifiable Disease Surveillance System (NDSS). Each case satisfied the Ministry of Health of the People’s Republic of Health malaria definition. Years lived with disability (YLD) estimated via DisMod II and Microsoft Office Excel 2003.	*Malaria cases and epidemiological burden from May to Oct 2007.* Cases 836 Epidemiological Burden *Flooding* **0-4 yrs:** 4, 0.036 YLD **5-14 yrs:** 9, 0.028 YLD *Waterlogging* **0-4 yrs:** 16, 0.194 YLD **5-14 yrs:** 50, 0.706 YLD	Intense flooding, as opposed to moderate rainfall, may disrupt mosquito breeding and prevent extensive malaria spread. Waterlogging on its own had more significant consequences for malaria than flooding.
**Boyce et al.** **(2016)** [24]		Quasi-experimental (difference-in-difference) Pre-flood: May 2012 to April 2013 Post-flood: June 2013 to May 2014	Children ≤15 in the Kasese District pre- and post-floods	Western Region, Uganda (Sub-Saharan Africa)	Kasese floods (May 2013)	Malaria hospitalisations and case differences	Hospitalisations referred to admissions at the Bugoye Level III Health Center (BHC). Malaria diagnosis confirmed through rapid testing at the hospital.	Hospitalisations *Pre-flood* **<5 yrs:** 211 | **5-15 yrs:** 137 *Post-flood* **<5 yrs:** 284 | **5-15 yrs:** 260 The pre-/post-flood difference in hospitalisations amongst 5-15 yrs was significant (*p* = 0.047).	Malaria transmission and hospitalisation increased in the post-flood as compared to pre-flood period. This was particularly true in villages bordering impacted rivers. The epidemic peaked three months after the May floods. This can be explained by mosquito breeding patterns.
**Elsanousi et al.** **(2018)** [25]		Descriptive: Surveillance Non-flood years: September-November of 2011 & 2012 Flood year: September-November of 2013	Children <5 in Sudan (Almanagil Locality) in non-flood vs flood years	Gezira State, Sudan (Sub-Saharan Africa)	2013 floods (August)	Malaria cases and incidence rates (per 100,000 person-days)	Cases had to meet the Sudanese Federal Ministry of Health’s definition of malaria. Cases reflect those captured by the sentinel malaria notification sites (SMNs) (9 hospitals and 4 health centres) and the Gezira State Ministry of Health.	Cases **SMNS** *Non-flood:* 11,032 (2011) & 11,917 (2012) *Flood:*10,603 (2013) **Confirmed (by hospitals)** *Non-flood:* 1,419 (2011) & 1,645 (2012) *Flood:* 2,307 (2013) Incidence Rates (95% CI) *Non-flood:* 9.80 (9.29, 10.32) in 2011 & 10.00 (9.52, 10.49) in 2012 *Flood:* 15.02 in 2013 Difference is statistically significant, *p* < 0.0001	The study concluded that the 2013 malaria epidemic was associated with flooding in the same year. Those <5 and especially those <1 were identified as the most at risk sector of the population.
**Liu et al.** **(2018)** [26]		Analytical: Time series Flood season (April to September), 2005-2012	Children <15 in Yongzhou in flood versus non-flood conditions	Hunan Province, China (East Asia & the Pacific)	12 floods, occurring during flood season of 2005-2012	Relative risk of floods on weekly typhoid cases	Weekly typhoid cases were reported by the National Notifiable Disease Surveillance System (NDSS) and utilised for analysis. Every case was clinically diagnosed and satisfied criteria set by the National Health and Family Planning Commission (NHFPC) of the People’s Republic of China.	Relative Risks (95% CI) *Calculated for lag0-4 (wks)* **0-4 yrs** 2.39 (1.02, 5.60)*, 1.01 (0.39, 2.62), 1.53 (0.65, 3.56), 1.40 (0.61, 3.23), 1.61 (0.66, 3.91) **5-14 yrs** 1.35 (0.81, 2.25), 1.23 (0.71, 2.13), 0.67 (0.35, 1.29), 1.09 (0.63, 1.89), 1.02 (0.57, 1.81) *p < 0.05	Floods likely led to increased consumption of water contaminated with diarrhoea-causing pathogens such as *Salmonella*. This study identified children <5 as a group at increased risk for typhoid post-flooding.
**Colston et al.** **(2020)** [27]		Analytical: Time series, cohort November 2009 to March 2014	Children ≤2 enrolled with the Santa Clara de Nanay site of MAL-ED project in early flood, late flood, and non-flood periods	Loreto, Peru (Latin America & the Caribbean)	2011-2012 La Niña-related flooding Early period: December 1, 2011 to February 29, 2012 Late period: March 1 to May 31, 2012	Rotavirus-positive stool samples and relative risk	MAL-ED participant stool samples were collected monthly or in the event of a diarrheal episode. Samples were tested for 14 pathogens (rotavirus being the only vaccine-preventable) through PCR and ELISA assays.	Samples *Number positive (% of all samples)* **Early flood:** 25 (2.9%) **Late flood:** 85 (10.2%) **Pre-/post-flood**: 173 (2.6%) Risk Ratio (95% CI) *Compared to non-flood periods & control sites* **Early flood:** not significant **Late flood:** 5.3 (2.70, 10.40), *p* <0.001	Having observed that children were 530% more at risk of contracting rotavirus in the late flood as compared to the pre-/post-flood periods (& control sites), the authors proposed vaccination as a disaster preparedness strategy in flooding-prone areas.
**Balikuddembe et al.** **(2023)** [28]		Analytical: Retrospective cohort 1990 to 2019	Children <5 in five East African countries of varying exposure to flooding	Ethiopia, Kenya, Somalia, Sudan, & Tanzania (Sub-Saharan Africa)	205 floods (2,869 days combined duration) between 1990 and 2019	Correlation between the number and duration of floods and malaria incidence	Flood data was extracted from EM-DAT, while malaria incidence reflected the Global Burden of Diseases Study (GBD) reporting.	Correlation Coefficient *Number of floods* **Ethiopia:** 0.264, *p* = 0.159 **Kenya:** -0.586, *p* = 0.001 **Somalia:** -0.421, *p* = 0.021 **Sudan:** -0.205, *p* = 0.278 **Tanzania:** -0.184, *p* = 0.331 *Duration of floods* **Ethiopia:** 0.294, *p* = 0.0317 **Kenya:** -0.657, *p* <0.0001 **Somalia:** -0.302, *p* = 1.039 **Sudan:** -0.420, *p* = 0.021 **Tanzania:** -0.457, *p* = 0.011	The findings suggest that the duration of floods is more influential on malaria incidence than the number of floods. The authors recommend that future studies consider the contribution of economic and sociocultural factors in the risks floods pose for health.
METEOROLOGICAL									
**Bhunia & Ghosh** **(2011)** [29]		Descriptive: Outbreak investigation May to August 2009	Children <15 admitted to a healthcare facility in Gosaba block No comparison (outbreak)	West Bengal, India (South Asia)	Cyclone Aila (May 25, 2009)	Acute watery diarrhoea cases and attack rates (per 10,000)	Cases were collected through monthly surveillance. To qualify as a case, patient had to be admitted to a healthcare facility for acute watery diarrhoea and severe dehydration. Random rectal swabs of those with acute watery diarrhoea were collected to determine source of outbreak.	Cases **0-4 yrs:** 124 **5-14 yrs:** 201 Attack Rate **0-4 yrs:** 54/10,000 (0.54%) **5-14 yrs:** 35/10,000 (0.35%) Sample Results 2/5 rectal swabs returned positive for *V.cholerae*. No pathogen was identified for the other 3.	The authors believe the pathogen *V.cholerae* was responsible for the 2009 diarrhoeal outbreak. Individuals likely became ill through drinking or handling contaminated water. Action was taken to manage the outbreak, including fixing the pipelines, chlorinating household water, and health education.
**Fredrik et al.** **(2015)** [30]		Descriptive: Outbreak investigation January 6-18, 2012	Children ≤14 in two localities of Pondicherry (total population: 8,367) No comparison (outbreak)	India (South Asia)	Cyclone Thane (December 29, 2011)	Cholera cases and attack rate (per 100,000)	Diarrhoea cases had to meet WHO guidelines within the study period. Cases were identified through door-to-door searching and healthcare facility reporting. Stool samples were randomly collected from patients admitted for diarrhoea. They were assessed for *Vibrio cholerae*, *Salmonella*, *Shiagella*, among other pathogens.	Cases **<5 yrs:** 76 **5-14 yrs:** 16 Attack Rate **<5 yrs:** 9/100,000 **5-14 yrs:** 11/100,000 Lab Results 9/16 rectal swabs came back positive for *V. cholerae*	Laboratory testing indicated that one strain of *V. cholerae* was to blame for the outbreak. The storm is believed to have caused the contamination of pipes delivering water for public consumption.
**Jones et al.** **(2016)** [31]	Descriptive: Outbreak investigation Pre-Tropical Depression: April 8 to July 14, 2013 Post-Tropical Depression: April 7 to July 13, 2014		Children <5 at the National Referral Hospital (NRH) in Honiara pre- and post-tropical depression	Guadalcanal, Solomon Islands (East Asia & the Pacific)	2014 Tropical Depression (March 31 to April 6)	Rotavirus cases and positivity rate	Faecal samples of those treated for diarrheal illnesses at the NRH were assessed for rotavirus.	Rotavirus Cases *Pre-Tropical Depression* #: 0 out of 12 Positivity rate: 0% *Post-Tropical Depression* #:18 out of 43 Positivity rate: 41.9% Difference in positivity rate is significant, *p* = 0.005	The authors believe rotavirus was largely responsible for the post-depression diarrhoeal outbreak, as no hospital samples were testing positive for rotavirus a year before the depression. However, the sample size was quite low. Most cases observed were amongst those under five.
**Wu et al.** **(2018)** [32]	Analytical: Case-crossover January 1983 to April 2009		Children <18 residing in Matlab and receiving treatment for cholera at the International Centre for Diarrhoeal Disease Research, Bangladesh (icddr, b) A case (hospitalised individual) was compared with itself 1 week before and after (control) hospitalisation	Bangladesh (South Asia)	Heatwaves between 1983-2009	Odds ratio for cholera during/after heatwave	A heat wave was defined as temperature in or above the 95^th^ percentile of the 30-year daily mean for 2+ consecutive days. Lag affect was studied for 5 days.	Odds Ratio (95% CI) **Heatwave:** 0.92 (0.74, 1.16), *p* = 0.498 **Heatwave 2-day lag:** 1.11 (0.88, 1.40), *p* = 0.387 **Heatwave 4-day lag:** 0.79 (0.64, 0.99), *p* = 0.036	The authors reported a positive association between heatwaves and cholera incidence when there was a 2-day lag. The study suggests that heat-associated increases in cholera may be due particularly to wet conditions.
**Li et al.** **(2021)** [33]	Analytical: Case-crossover June to October, 2013-2018		Children <18 in nine cities of Pearl River Delta A case (dengue fever onset day) was matched to 3-4 controls (the same day of the week throughout the month)	Guangdong Province, China (East Asia & the Pacific)	20 tropical cyclones between 2013 and 2018 Cyclones, by year **2013:** 3, **2014:** 3 **2015:** 2, **2016:** 3 **2017:** 5, **2018:** 4	Dengue fever cases and relative risk	The data came from the National Notifiable Disease Surveillance System (NDSS). Upon diagnosis, dengue fever must be reported to local health authorities swifty (within a day).	Cases, by year **2013:** 227, **2014:** 3,921 **2015:** 1, **2016:** 2 **2017:** 84, **2018:** 173 Relative Risk (95% CI) *Exact RRs not given, apart from the strongest* **Typhoon, lag 7:** 1.6 (1.13, 2.28)	The study concluded that an associated risk does exist when it comes to tropical cyclones and dengue fever. However, age did not seem to be a strong predictive factor.

**Table 3:** Summary of Studies on Association between Disasters and Disruption of Immunisation Services by Family of Event, Ordered by Year of Publication.

Author & Year	Study Design & Period	Population, Sample & Comparison	Setting & Region	Disaster	Vaccine(s)	Analysis & Methods	Results	Conclusions
GEOPHYSICAL								
**Tohme et al.** **(2017)** [34]	Ecological 2009–2015 Pre-earthquake: 2009 Earthquake year: 2010 Post-earthquake: 2011–2015	12-month-olds in Haiti pre- and post-earthquake	Haiti (Latin America & the Caribbean)	2010 Haiti earthquake (January 12^th^, 2010)	DTP3^1^/Penta3^2^, OPV3^3^ & MCV^4^	WHO-reported immunisation coverage. Vaccine coverage was monitored as three doses for DTP3/Penta3 and OPV3 and one dose for measles.	Coverage **MCV** *Pre*: 60% *Earthquake yr*: 45% *Post*: 58%, 69%, 80%, 64%, 64% **DTP3/Penta3** *Pre*: 68% *Earthquake* yr: 69% *Post*: 85%, 85%, 85%, 60%, 72% **OPV3** *Pre*: 65% *Earthquake yr*: 62% *Post*: 79%, 80%, 92%, 75%, 76%	One of the main priorities following the earthquake was to bolster the national immunisation services in recognition of the risk the event presented for VPD outbreaks. Campaigns MCV: February-June 2010 MCV and OPV: 2012 MR and OPV: March-April 2016 In order to maintain progress, efforts should be made to manage without external funding and navigate priorities concerning vaccination.
**Ahmad et al.** **(2018)** [35]	Analytical: Retrospective, cohort January 2011 to October 2016 Pre-earthquake: Jan 2011 – Sep 2015 Post-earthquake: Nov 2015 – Oct 2016	Children under 12 months in ten districts of Khyber Pakhtunkhwa province pre- and post-earthquake	Pakistan (South Asia)	October 2015 Hindu Kush earthquake (October 26^th^, 2015)	Number of patients obtaining measles or pentavalent^2^ vaccine or full immunisation	Data was collected from the District Health Information System (DHIS). Ten earthquake-affected districts were evenly split into Group I and Group II.	The study noted a significant dip in the number of children fully immunised in the month of the earthquake. However, the numbers quickly returned to normal. Example *Group 1, full vaccination estimates* **September 2015:** 18,000 **October 2015:** 13,000 **November 2015:** 17,000 **December 2015:** 20,000	The authors attributed this drop in coverage to the immediate earthquake-related disruption of health services. However, they also mentioned that the work of humanitarian organisations is meant to help avert this gap in immunisation.
**Thapa et al.** **(2020)** [36]	Mixed method: Descriptive analysis & qualitative (focus groups) August to December 2017	Children from 29 Village Development Committees in nine districts pre- and post-earthquake	Nepal (South Asia)	April 2015 Nepal earthquake (April 25^th^, 2015)	BCG^5^, DPT-HepB-Hib1^6^, DPT-HepB-Hib3^7^, polio 1, polio 3, and MR^8^ coverage	Data was extracted from Health Management Information System	Coverage *Pre- vs post-earthquake* **BCG:** 71.4% vs. 61.9% **DPT-HepB-Hib1:** 79.2% vs. 78.9% **DPT-HepB-Hib3:** 77.5% vs. 77.9% **Polio 1:** 77.2% vs. 77% **Polio 3:** 77.6% vs. 77% **MR:** 86.1% vs. 80.1%	Health services were largely able to be carried out post-earthquake through makeshift clinics. Vaccination rates may have been sustained in part thanks to MR campaigning.
**Khanal** **(2022)** [37]	Analytical: Difference-in-difference 2011 versus 2016	Children <5 in 12 treated and 23 control districts, pre- and post-earthquake	Eastern, Central and Western Regions of Nepal (South Asia)	2015 Gorkha earthquake (April 25^th^, 2015)	Diff-in-diff estimate between timely immunisation pre- and post-earthquake	Data was sourced from the 2011 and 2016 Nepal Demographic Health Surveys. Outcomes for two age groups were considered: < 1 and <5 years.	Timely Immunisation *Diff-in-diff estimate (SE)* **<1 yr:** -0.116 (0.076) **<5 yrs:** -0.088* (0.044) *statistically significant, *p* < 0.05	The decrease in timely vaccination is believed to be mainly an issue of supply. Many clinical spaces were impacted by the earthquake.
METEOROLOGICAL								
**Colón-Lopez et al.** **(2021)** [38]	Analytical: Retrospective, cohort 2015–2019 Pre-hurricane: July 2015 to August 2017 Post-hurricane: October 2017 to December 2019	Children ages 11–17 in Puerto Rico pre- and post-hurricane	Puerto, Rico United States (Latin America & the Caribbean)	Hurricane Maria (September 2017)	HPV^9^, Tdap^1^ & MenACWY^10^ initiation rates	Data was obtained from the PR Immunization Registry. 87% of Puerto Ricans are represented in this registry.	In the month of Hurricane Maria (September 2017), the initiation rate for HPV dipped from 85% the previous month to 75%. The MenACWY and Tdap vaccines experienced a less dramatic (about 5%) but notable decrease in initiation.	The immunisation initiation rates for the three vaccines took about three months to recover. The authors believe the more severe drop in the post-hurricane initiation rate of HPV as opposed to meningococcal and Tdap reflects sentiments around the vaccines.
**Fernandes et al.** **(2022)** [39]	Analytical: Interrupted time series analysis November 2016 to March 2020	Children <5 in 25 districts of Manica and Sofala provinces pre- and post-disaster	Mozambique (Sub-Saharan Africa)	Cyclone Idai (March 2019)	Relative immunisation loss (measles, BCG^5^, DPT-Hib3^11^, and full) in month of cyclone (March 2019) and two months following (April and May)	Immunisation data was obtained from the Sistema de Informação de Saúde para Monitoria e Avaliação. The relative loss was the ratio of monthly observed to expected counts (estimated using generalized linear mixed model).	Relative Loss (95% CI) *March, April, May 2019* **Measles:** 0.87 (0.67, 1.14), 0.87 (0.61, 1.29), 1.10 (0.97, 1.25) **BCG:** 0.90 (0.71, 1.14), 0.92 (0.67, 1.29), 1.10 (1.00, 1.21) **DPT-Hib3:** 0.92 (0.72, 1.18), 0.87 (0.64, 1.23), 1.06 (0.96, 1.18) **Full:** 0.86 (0.66, 1.12), 0.88 (0.63, 1.27), 1.07 (0.96, 1.20)	The hit to vaccination post-Idai may have been mitigated by international/domestic aid and a resilient healthcare system. The authors, however, warn that although data seems to suggest that vaccine services recovered by May 2019, there is a need to compensate for the affected months.
CLIMATOLOGICAL								
**Nagata et al.** **(2021)** [40]	Analytical: Retrospective analysis 2011–2019	Children in 22 sub-Saharan African countries born during drought vs non-drought conditions between 2011 and 2019 (N = 137, 379)	Sub-Saharan Africa	Drought between 2011 and 2019	Risk difference for BCG^5^, DPT^1^, polio, and measles immunisation when born in drought vs non-drought conditions	Data was borrowed from Demographic and Health Surveys. Vaccination status was measured in the surveys by vaccination card or mother’s reporting.	Risk Difference (95% CI) *Adjusted* **BCG:** -1.5 (-2.2, -0.9) **DPT:** -1.4 (-2.2, -0.5) **Polio:** -1.3 (-2.3, -0.3) **Measles:** -1.9 (-2.8, -0.9)	Children born during a drought were at increased risk of not receiving their immunisations at birth or before their first birthday. The authors suggest there may be a difference in acquisition for babies born in hospital versus at home.

1 Diphtheria, tetanus and pertussis vaccine

2 Diphtheria, tetanus, pertussis, hepatitis B (hepB), and *Haemophilus influenzae* type B (Hib) vaccine

3 Oral polio vaccine

4 Measles-containing vaccine

5 Bacillus Calmette-Guérin vaccine for TB

6 Diptheria, tetanus, pertussis, polio, Hib, and hepB vaccine, first dose

7 Diptheria, tetanus, pertussis, polio, Hib, and hepB vaccine, third dose

8 Measles and rubella vaccine

9 Human papilloma virus vaccine

10 Meningococcal conjugate vaccine

11 Diphtheria, tetanus, pertussis, and *Haemophilus influenzae* type B (Hib) vaccine

**Fig 2:**
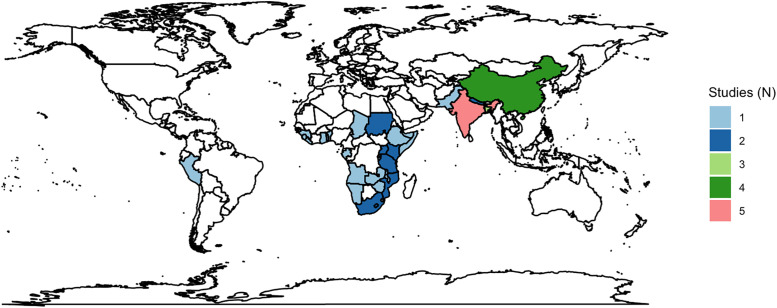
Map of number of studies corresponding to each country.

The literature covered several regions, with 11 of the 26 studies concentrating on World Bank-classified South Asia, six on Sub-Saharan Africa, five on East Asia & the Pacific, and four on Latin America & the Caribbean region (See Fig 2). Balikuddembe et al. and Nagata et al. considered a respective five and 22 African countries each [28,40]. No studies included in this review emerged from Europe and Central Asia, the Middle East and North Africa, or North America. India and China were the most examined countries, having been the focus of a respective five and four studies each. Of the study countries, 13 were deemed low-income, 15 lower-middle income, five upper-middle income, and one high income by 2025 World Bank classification [41]. All studies were published in English.

We identified a wide range of disasters in the literature. Nine studies each looked at IRDR-classified geophysical and hydrological disasters. While seven focused on meteorological disasters and one a climatological disaster. All the hydrological disasters considered were floods. The other disaster events that garnered the most attention were earthquakes and cyclones, mentioned in nine and six studies, respectively. Two of the earthquake studies looked at the 2010 Haiti earthquake, while both tsunami studies covered the 2004 Indian Ocean tsunami. Finally, droughts and heatwaves were the events at the centre of one study each. Notably, we did not find any studies on the effect of wildfires and volcanic eruptions.

**Fig 3:**
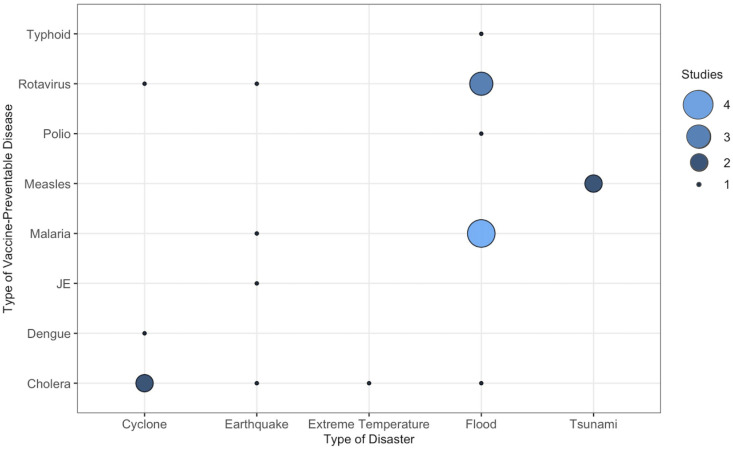
The effect of disaster type on vaccine-preventable disease and (B) vaccine disruption. Disasters were classified as IRDR main events (See S1 Fig). The 2004 Indian Ocean earthquake and tsunami was categorised as a tsunami and tropical depressions and hurricanes were considered cyclones for the purpose of this graph.

**Fig 4:**
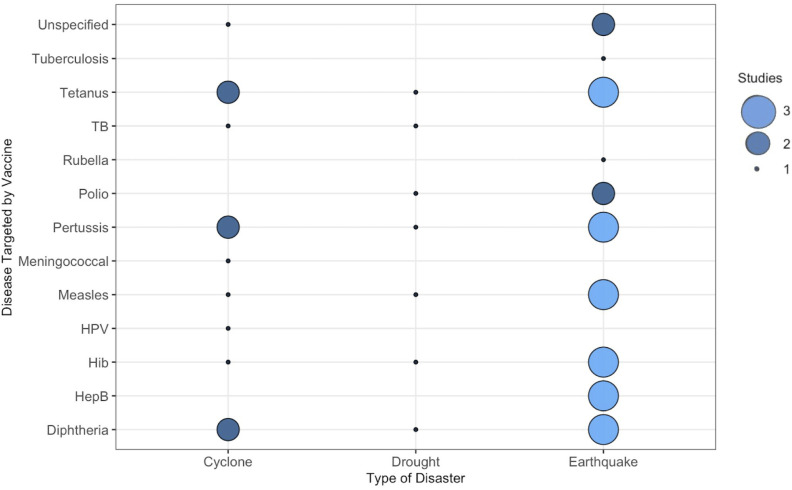
The effect of disaster type on vaccine disruption. Disasters were classified as IRDR main events (See S1 Fig). The 2004 Indian Ocean earthquake and tsunami was categorised as a tsunami and tropical depressions and hurricanes were considered cyclones for the purpose of this graph.

Out of the 26 studies included, 19 concerned VPD outbreaks (See Table 2). These studies directly addressed outbreaks of typhoid, rotavirus, polio, measles, malaria, Japanese encephalitis (JE), dengue, and cholera (See Fig 3 and Fig 4). Three studies reporting on diarrhoeal outbreaks attributed cases to cholera (Bhunia & Ghosh), rotavirus (Karmarkar et al.) or a combination of the two (Schwartz et al.). The included studies used different study designs for evaluating the impact of hazards on vaccine-preventable diseases, which included outbreak investigations (N = 7), descriptive analyses of surveillance data (N = 3), case-crossover (N = 3), time series regressions (N = 3), retrospective cohorts (N = 3), amongst others.

Seven studies evaluated the impact of disasters on vaccination, including vaccines against measles [34–36,39,40], hepatitis B [34–36], diphtheria [34–36,38–40], tetanus (34–36 & 38–40, polio [34,36,40], pertussis [34–36,38–40], *Haemophilus influenzae* type B [34–36,39], tuberculosis [36,39,40], rubella [36], human papillomavirus [38], and meningococcal [38]. Three studies looked at full [35,39] or timely [37] immunisation without defining the vaccines involved.

Amongst the studies included in this review, none explicitly measured the effect of a disaster on the vaccination rate of any of the following thirteen WHO recommended immunisations: pneumococcal, rotavirus, JE, yellow fever, tick-borne encephalitis, typhoid, cholera, hepatitis A, rabies, dengue, malaria, influenza, or varicella.

### Investigation of outbreaks by social and demographic factors

Eleven studies explicitly concentrated on the vaccination status and/or VPD cases amongst children under and/or inclusive of five. Discussion of routine immunisation for this age group addressed vaccines protecting against meningitis, diphtheria, tetanus, polio, tuberculosis, amongst full vaccination for age [34,35,37,39]. Of the 19 disaster-related VPD outbreak studies, seven reported attack rates or relative risks amongst those five and younger [16,19,21,25,27,28,31].

After the Kashmir earthquake, Karmakar et al. noted high rotavirus infections amongst infants [16]. The authors indicated that this age group was less likely to have directly consumed the contaminated water (a source of the outbreak), postulating that contraction may have resulted from contact with a care provider [16]. Although measles typically affects children under five, Mohan et al.’s study on post-tsunami outbreak of measles in India found that 36% of the cases occurred in individuals over five years [15]. Balasubramaniam & Roy similarly examined the measles outbreak in Tamil Nadu post the 2004 Indian Ocean earthquake and tsunami, noting that the measles vaccine is only prescribed in the area up to a child’s first birthday [17].

Along with age, race and socioeconomic status were cited as factors influencing a child’s susceptibility to VPDs in a disaster context. In an outbreak of 412 polio cases in South Africa post-flooding, only seven cases were amongst non-Black children [21]. However, rates by race-ethnicity were not described in this paper [21]. In a 2006 study of diarrhoea amongst Bangladeshi children during flooding, the authors remarked that refugees are an especially susceptible population when it comes to falling ill during a cholera outbreak [22], but without providing specific comparisons between refugee and non-refugee children. Schwartz et al. further recognised proxies of low socioeconomic status (i.e., father not formally educated) as a risk factor for contracting cholera post-flood [22].

### Impacts of floods on WASH and vector-borne VPDs

Almost a third of the studies included considered flooding events (N = 9, 20–28). Another six studies focused on cyclones [29–31,33,38,39], which commonly induce flooding.

After Cyclone Aila in India, an outbreak investigation found a 0.54% attack rate of cholera amongst children under-5, possibly due to contaminated communal water sources [29]. Schwartz et al. likewise discussed the circulation of cholera via the faecal-oral pathway during flood times in Bangladesh [22]. Based on data from the International Center for Diarrheal Disease Research, Bangladesh (ICDDR, B), the proportion of daily diarrhoea cases amongst patients under 15 attributed to *V. cholerae* was 37% during flood versus 20% in non-flood periods [22].

Many authors pointed out that floods can also lead to people consuming food more often that has been handled or prepared with unpurified water. Uddin Ahmed et al. cited poorly prepared food as a source of rotavirus post-monsoon [20]. Liu et al. additionally cited the consumption of faecal matter due to untreated or improperly treated water in flood areas of Yongzhou, China as the primary cause of typhoid contraction in the years studied [26]. The authors found that children under 5 exposed to flood conditions had a 2.39-fold higher risk of developing typhoid at lag0 compared to those not exposed to flooding between 2005 and 2012 [26].

Vector-borne diseases, such as malaria, were commonly observed in studies of flooding [23–25,28]. Post-flood patterns in malaria incidence are thought to reflect mosquito breeding timing. Malaria epidemic peaked in the Ugandan Highlands three months post-flooding, likely because vector habitats are initially wiped away and then re-established in ideal conditions [24]. Post-flood malaria hospitalisations of those 5–15 years old were up 123 from pre-flood hospitalisations (260 vs 137, p = 0.047) [24]. However, another study suggested that malaria burden in children aged 14 and younger is likely higher due to waterlogging than flooding [23]. Another study suggested that the duration rather than the number of floods had the greater impact on malaria cases amongst children under 5 in East African countries [28]. Relatedly, Elsanousi et al. reported a difference in the incidence rates of malaria in children under five in Sudan in non-flood versus flood years (p < 0.0001) [25].

### Disaster-induced obstacles to routine childhood immunisation

Seven of the 26 studies either focused on or mentioned vaccine disruption after a disaster (34–40, See Table 3). Regarding the impact of Hurricane Maria on the adolescent uptake of HPV, Tdap, and meningococcal vaccine in Puerto Rico, Colón-López et al. found the disaster to have the most substantial effect on HPV vaccination, leading to an almost instant 15% reduction in initiation rate (90% to 75%) during the month of the event [38]. The authors suggested the hurricane caused significant infrastructural and electrical issues, leading clinics to close for repair and vaccines to expire [38].

Ahmad et al. [35], Khanal [37], and Thapa et al. [36] examined the impact of major earthquakes on childhood vaccination. One study observed a temporary decline in full immunisation among children under one following the 2015 Hindu Kush earthquake, likely due to service disruptions, though rates quickly returned to normal with humanitarian intervention [35]. Similarly, after the 2015 Gorkha earthquake in Nepal, there was a 9% drop in timely vaccination among children under five, which was attributed to vaccine supply issues, cold chain disruptions, lack of medical personnels, and suspended healthcare services [37]. A second study analysing immunisation coverage before and after the Gorkha earthquake in Nepal focused on BCG, DPT-HepB-Hib, polio, and MR vaccines. BCG coverage saw the largest decline, from 71.4% to 61.9%, though the study does not explore causes in depth [36].

Health infrastructure damage and issues with vaccine storage were also cited in retrospective studies looking at droughts between 2011 and 2019 in sub-Saharan Africa [40], with significantly lower vaccine coverages of BCG, DTP, Polio and measles among children in 22 sub-Saharan African countries born during drought vs non-droughts. The authors remarked that drought could introduce financial stress or provoke migration making healthcare inaccessible and vaccination, consequently, unattained [40].

In the wake of Cyclone Idai in Mozambique, Fernandes et al. remarked that water and electrical services were disrupted, and health facilities suffered structural devastation, resulting in temporary decreases in childhood immunisation coverage, particularly in the Sofala and Manica provinces [39]. A cholera outbreak in Sofala shortly after the cyclone may have additionally overwhelmed the healthcare system, limiting services available [39].

Amongst the studies specifically concerned with disaster-related vaccine disruption, damage to health facilities and problems with vaccine storage emerged as the most discussed contributors to reduced immunisation [37–40]. Financial instability and migration were additionally recognized as potential post-disaster obstacles to vaccination [40]. As suggested by Nagata et al., there remains a need to study specific factors brought on by disaster that restrict access to and reception of routine vaccines [40].

## Discussion

Our review contributes to the current knowledge on the effects of disasters on vaccination and VPDs, summarising and illustrating the risks these events pose to children’s health. Although relevant publications are becoming increasingly frequent, suggesting that disaster-associated VPD outbreaks and vaccine disruption are gaining more attention, there is still limited knowledge about the threat that disasters pose to VPD outbreaks and childhood immunisation services. Our review is critical, as disasters create conditions conducive to VPD outbreaks, especially in low-resource settings and among socially vulnerable populations and communities, who can be particularly affected by weak WASH structures. These events can further disrupt routine immunisation, particularly when health systems are fragile. In addition, our review identified a lack of quantitative evidence on the effects of hydro-meteorological and geological disasters, especially among the most socially vulnerable children, both in high- and low- and middle-income countries.

Historically, low- and middle-income countries (LMICs) have suffered the brunt of disasters, with six out of ten of the top countries experiencing these events (between 1995–2024) belonging to this region [42]. Notably, 28 out of the 34 countries amongst the studies of this review fell into the low-income and lower-middle income categories defined by the World Bank in 2025 [41]. Poverty and inequality, more prevalent in LMICs, signify that large populations live below adequate living standards and in poor infrastructure, making them more susceptible to hydro-meteorological and geological disasters, including compound hazards [43]. It is key to establish a baseline level of vaccination in contexts with combined vulnerability to extreme weather events and fragile systems to effectively respond to them, especially as these events are likely to continue if not worsen due to climate change [42].

Equity issues regarding vaccination exist both within and between countries. Globally, there remain inequities in vaccine distribution, resulting in differences in global inequalities in susceptibility to disaster-related VPD outbreaks. For example, by January 2022, most of the world was immunised against COVID-19 (two-thirds) yet under 10% of those residing in low-income countries had been vaccinated [44]. A vaccine tax, entailing wealthy countries to divert a portion of the money reserved for vaccines to a fund (i.e., COVAX) for low-income countries [45], could help eliminate disparities in childhood vaccination rates globally, likely reducing the number of post-disaster VPD outbreaks in low-income nations.

Our review highlights that the differential impacts of disasters on children from different socioeconomic backgrounds have been poorly studied. It is important that disaster studies provide the impacts on specific sociodemographic groups to better understand high-risk populations during VPD outbreaks. In high and low- and middle-income countries alike, specific populations may not be as highly vaccinated as others, leading to higher burdens of VPDs amongst some communities more than others post-disaster. For instance, a study between 2010 and 2019 found 35 of 64 analysed countries to have significant differences in DPT vaccination rates amongst children of distinct ethnic groups aged between 12–29 months [46]. In the Philippines, over 50% of the Maranao ethnic group was not vaccinated against DPT, as opposed to 8% of the largest ethnic group (Tagalog) [46].

Disaster-related VPD outbreaks may be avoided, in part, by addressing racial and ethnic disparities in childhood vaccination rates. For instance, Van Middlekoop et al.’s 1987/88 South African outbreak study shared that seven out of 412 polio cases in Natal/KwaZulu were amongst non-Black children [21], despite the 1980 census detailing a racial breakdown of 49.1% Black, 25.5% Indian, and 22% White (at the time of study) [47]. If the polio cases were truly representative, 210 of them would be amongst Natal/KwaZulu’s Non-Black population. However, it is worth noting this study was held during the Apartheid.

Despite minimal attention from the studies included in our review, malnutrition can further be exacerbated in disaster time and affect children’s susceptibility to infection [48]. Poor nutritional status and infection can be cyclical in nature - malnutrition invites infection and infection may bring about malnutrition [49]. Vaccination partially breaks this cycle, protecting malnourished populations from preventable infections. Malnutrition is particularly discussed in relation to drought, as was the case with Nagata et al. [40]. A drought may not be as immediately impactful when it comes to population health as a tsunami or earthquake, but it may give rise to food scarcity and insecurity which can eventually lead to elevated rates of malnutrition and susceptibility to infection [40]. Ideally, disaster responses prioritise food and water security alongside continued and emergency vaccination efforts [40,48].

While not considered in-depth in the studies of our review, if coinciding with recent conflict or displacement events, the effect of disaster on VPD spread and routine immunisation could amplify already existing health inequalities. Of note, only one of our papers specifically focused on a conflict setting. In Mozambique, Cyclones Idai and Kenneth came at a time of civil unrest, specifically armed conflict in regions such as Sofala [50]. This study suggested that combined tragedies increased population displacement and forced internal and external migration and the interruption of healthcare services [50]. Conflicts can additionally leave a population quite vulnerable to disaster-related VPD outbreaks, as the healthcare system may be weakened, the population under-vaccinated, and the government unprepared to control the spread. Notably, the United Nations Children’s Fund (UNICEF) reports that close to 40% of unvaccinated or under-vaccinated children globally reside in countries experiencing conflict [51]. Further research could investigate the extent to which disasters exacerbate pre-existing health inequalities resulting from conflict.

Countries should be prepared to carry out emergency vaccination campaigns when the threat of an outbreak exists [52]. A previous systematic review regarding vaccination guidance in crisis settings found most of the documents surveyed contained recommendations for measles, polio, and tetanus vaccines [53]. Whilst this review was not specific to climate-induced disasters, it does shed light on vaccinations prioritised in resource- and time-limited settings. The WHO’s “Vaccination in Humanitarian Emergencies” guideline outlines the steps necessary to immunise a population against a disease with outbreak potential, suggesting the creation of an Immunization Task Force (ITF) [54]. In the case of a disaster, the ITF determines which vaccines are to be administered and performs a situation analysis before administering immunisations [54]. Whilst these guidelines provide a useful framework in crisis situations, it is uncertain to what extent they are followed in each country.

Amongst the disasters covered in our review, floods gained particular attention for introducing or exacerbating unsafe WASH and influencing vector population dynamics, thereby facilitating the spread of vaccine-preventable pathogens. It is worth noting that the literature on flooding and diarrhoeal diseases is extensive; however, many of these studies did not meet our inclusion criteria, as they were not specific to VPDs or children. Floods have been known to cause the spread of diarrhoea-causing vaccine-preventable (i.e., *V. cholerae*) and non-vaccine-preventable (i.e., *Escherichia coli*) bacterial infections [55]. In addition to routine and supplementary vaccination, diarrhoeal outbreaks can be controlled post-disaster through good community hygiene practices, such as consuming and utilising safe water, adequate handwashing, and not defecating in bodies of water [56].

Disasters not only present opportunities for VPD outbreaks in insufficiently vaccinated populations but can also disrupt existing vaccination efforts. Our review summarised some of the obstacles to routine vaccination post-disaster, including infrastructural damage to immunisation sites, vaccine supply and demand issues, and introduced distance and lack of familiarity with the healthcare system due to displacement. Although our review contributes to measures that can be put in place to ensure healthcare systems are prepared to carry out immunisation programmes amidst the turmoil of a disaster, ultimately, achieving high vaccine coverage pre-disasters could mitigate disasters-related risks. In addition, the ability of a country to carry on with or experience minimal pausing in childhood vaccination services in the wake of a disaster speaks to the resilience of its healthcare system. A delay in routine immunisation following a disaster is largely expected, as traumatic injuries and illnesses take precedence from a healthcare perspective [57]. However, the quick resumption of vaccination activities demonstrates incredible forethought and planning. It is additionally worth considering the seasonality of climate hazards when scheduling vaccination. For instance, in some countries, the flu vaccination period overlaps with hurricane seasons [58]. While it may be difficult to shift immunisation timelines due to vaccination development processes, disaster-prone areas should make plans to ensure the continuation of vaccination efforts in anticipation of extreme weather events, especially as they are expected to increase in frequency and intensity with climate change [59].

Evidence indicating vaccination is more critical now in light of climate change than ever before comes at a time when vaccine hesitancy is a major threat to global health [60,61]. The prevalence of VPDs is expected to increase yet American states are removing their vaccines mandates in schools and places of employment [60,62], which inspires deep concern from public health experts taking into consideration the frequency of hydro-meteorological and geological disasters in some states [62].

### Strengths and limitations

Our scoping review has several strengths, such as including articles published in either English, Spanish, or Portuguese, without time restrictions, and including all the current (as of January 2025) WHO-recommended vaccines for children in the analysis. We recognise that WHO-recommended vaccines were added to the list at varying points in time and studies, thus, some studies may have been published prior to their inclusion in the list. Initially, the WHO’s Essential Programme on Immunisation (established in 1974) focused on vaccinating children against six diseases: Bacillus Calmette-Guérin (BCG), diphtheria, pertussis, tetanus, polio, and measles [63]. Today, this list has grown to twelve, adding Haemophilus influenzae type B (Hib), Hepatitis B (HepB), rubella, pneumococcal disease, rotavirus, and human papillomavirus (HPV) [63]. Including studies prior to and following these additions may permit insight into the health benefits of these vaccines when disaster strikes.

Despite the strengths of our study, it is not without its limitations. Firstly, although numerous languages were considered, the search terms were entered into the databases in English, which may have excluded literature not indexed in English. Secondly, many types of disasters were not represented in the literature despite their known impact on child health. Notable omissions include tornadoes, landslides, volcanoes, and wildfires. Currently, a great deal of consideration has gone to the impact of wildfires on children’s respiratory health [64]. As with any disaster, however, wildfires disrupt health services, posing potential obstacles to timely vaccine delivery. Third, we did not compile evidence on the effect of disasters on generalised diarrhoea, despite it being commonly discussed in the literature in relation to VPDs. The reason for this is that apart from a few diarrhoea-causing viruses (i.e., rotavirus) and bacteria (i.e., cholera), many diarrhoeal cases cannot be prevented through immunisation [65]. Fifth, this review does not account for the differences in magnitude of the included disasters, which may have influenced the extent to which these disasters impacted disease outbreaks and vaccination efforts. Finally, in covering a diverse range of disasters, geographic contexts, and vaccine-preventable diseases and their affiliated vaccines, this review inherently prioritizes scope over detail. Lastly, the criteria for the studies to be published may have meant the research largely came from high-income nations, thereby limiting generalisability.

## Conclusions

This scoping review summarises the growing yet still limited global literature on the effects of hydro-meteorological and geological disasters on VPDs and routine immunisation among children. The studies considered spoke to the capacity of disasters to give rise to VPD outbreaks and disrupt ongoing vaccination efforts. Certain populations, namely young children and refugees, were identified as vulnerable groups for contracting a VPD post-disaster, either because of low pre-disaster vaccination rates or facing the burden of its impacts. However, the magnitude of differential effects by socioeconomic characteristics was infrequently studied. Floods were considerably covered in the literature, as they commonly facilitate the spread of water-borne and vector-borne VPDs. Moreover, disasters may cause infrastructural damage and healthcare system disorder, disrupting routine childhood immunisation.

There is a need for studies examining the effects of disasters on VPDs absent from this review such as influenza and yellow fever, and vaccinations recently introduced as routine such as malaria and dengue. Additionally, further research should investigate the impact of disasters like wildfires and volcanic eruptions on VPD outbreaks and routine childhood vaccination.

As underscored in this review, vaccination alone is not enough to protect populations from disease post-disaster. WASH measures should accompany vaccination, and healthcare systems must build resilience and be prepared to provide non-acute services post-disasters. Addressing disparities in national and global vaccination efforts and building resilient health systems will help to reduce the threat of VPD outbreaks and vaccination disruption post-disaster. Vaccination should be a recognised component of disaster preparedness and incorporated into national and local policies accordingly.

## Supporting information

S1 PRISMA Checlist PRISMA Checklist. Preferred Reporting Items for Systematic reviews and Meta-Analyses extension for Scoping Reviews (PRISMA-ScR) Checklist (2018).
(DOCX)

S1 Table Medline search. Table formatting inspired by Walika et al.’s systematic review, “Outbreaks Following Natural Disasters: A Review of the Literature” [61].
(DOCX)

S2 Table EM-DAT Registration. EM-DAT records were examined for inclusion of disasters in the studies.
(DOCX)

S1 Fig IRDR disaster classification. (DOCX)
