# Supplementary material for: Effects of hydro-meteorological and geological disasters on vaccine-preventable disease outbreaks and routine immunisation amongst children: A scoping review
Source: PLOS Glob Public Health. 2026 Jun 12;6(6):e0005712. doi: 10.1371/journal.pgph.0005712 (PMC13262859; doi:10.1371/journal.pgph.0005712)
Supplement: S1 Table — Table formatting inspired by Walika et al.’s systematic review, “Outbreaks Following Natural Disasters: A Review of the Literature” [61]. (DOCX) [file pgph.0005712.s002.docx]

**S1 Table.** *Medline search*. Table formatting inspired by Walika et al.’s systematic review, “Outbreaks Following Natural Disasters: A Review of the Literature” (62).

| **Database** | **Concept** | **Search Terms** |
| --- | --- | --- |
| Medline | Vaccines (#1) | Vaccination* OR vaccinate OR immuni?ation* OR immuni?e OR vaccine* OR BCG vaccine OR Bacillus Calmette-Guerin vaccine* OR Hepatitis B vaccine* OR polio vaccine* OR DTP-containing vaccine* OR DTwP vaccine* OR DTaP vaccine* OR Tdap vaccine* OR DT vaccine* OR Td vaccine* OR Haemophilus influenzae type b vaccine* OR Hib vaccine* OR pneumococcal vaccine* OR rotavirus vaccine* OR MMR vaccine* OR measles vaccine* OR rubella vaccine* OR HPV vaccine* OR human papillomavirus vaccine* OR Japanese encephalitis vaccine* OR yellow fever vaccine* OR tick-borne encephalitis vaccine* OR typhoid vaccine* OR cholera vaccine* OR meningococcal vaccine* OR MenB vaccine* OR MenACWY vaccine* OR Hepatitis A vaccine* OR rabies vaccine* OR dengue vaccine* OR malaria vaccine* OR mumps vaccine* OR influenza vaccine* OR flu vaccine* OR flu shot* OR varicella vaccine* OR chickenpox vaccine* |
|  | Vaccine-preventable diseases (#2) | Vaccine-preventable disease* OR tuberculosis OR TB OR leprosy OR Hansen's disease OR Buruli ulcer OR Hepatitis B OR polio OR poliomyelitis OR diphtheria OR tetanus OR pertussis OR whooping cough OR haemophilus influenzae type b OR Hib OR pneumococcal disease* OR streptococcus pneumonia OR rotavirus OR measles OR rubeola OR rubella OR human papillomavirus OR HPV OR Japanese encephalitis OR yellow fever OR tick-borne encephalitis OR typhoid OR typhoid fever OR enteric fever OR cholera OR meningococcal OR Hepatitis A OR rabies OR dengue OR malaria OR mumps OR influenza OR flu OR varicella OR chickenpox |
|  | Disasters (#3) | Disaster* OR natural disaster* OR earthquake* OR mass movement* OR rockfall* OR mudflow* OR landslide* OR rotational slip* OR volcanic activity OR volcanic eruption* OR flood* OR tsunami* OR storm surge* OR convective storm* OR thunderstorm* OR hurricane* OR tornado OR tornadoes OR extratropical storm* OR blizzard* OR Nor'easter* OR extreme heat OR extreme cold OR heatwave* OR cold front* OR fog OR smog OR tropical cyclone* OR typhoon* OR cyclonic storm* OR drought* OR glacial lake outburst* OR wildfire* |
|  | Children (#4) | Child or children OR kid* OR infant* OR baby OR babies OR girl* OR boy* OR teen* OR teenager* OR adolescent* OR newborn* |
| Combined search: #1 OR #2 AND #3 AND #4 | | |
