# Supplementary material for: Effects of hydro-meteorological and geological disasters on vaccine-preventable disease outbreaks and routine immunisation amongst children: A scoping review
Source: PLOS Glob Public Health. 2026 Jun 12;6(6):e0005712. doi: 10.1371/journal.pgph.0005712 (PMC13262859; doi:10.1371/journal.pgph.0005712)
Supplement: S2 Table — EM-DAT records were examined for inclusion of disasters in the studies. (DOCX) [file pgph.0005712.s003.docx]

**S2 Table.** *EM-DAT Registration.* EM-DAT records were examined for inclusion of disasters in the studies.

| **Study** | **Location** | **Disaster** | **EM-DAT** |
| --- | --- | --- | --- |
| Mohan et al. | Tamil Nadu, India | 2004 Indian Ocean earthquake and tsunami |  |
| Karmakar et al. | Kashmir, India | 2005 Kashmir earthquake |  |
| Balasubramaniam & Roy | Tamil Nadu, India | 2004 Indian Ocean earthquake and tsunami |  |
| Zhang et al. | Gansu Province, China | 2008 Wenchuan earthquake |  |
| Barzilay et al. | Haiti | 2010 Haiti earthquake |  |
| Uddin Ahmed et al. | Mymensingh, Bangladesh | 1988 flooding |  |
| Van Middlekoop et al. | Natal/KwaZulu, South Africa | 1987 South Africa floods |  |
| Schwartz et al. | Dhaka, Bangladesh | 1988 flood |  |
|  |  | 1998 flood |  |
|  |  | 2004 flood |  |
| Ding et al. | Anhui Province, China | 2007 Huaihe River floods |  |
| Boyce et al. | Western Region, Uganda | Kasese floods |  |
| Elsanousi et al. | Gezira State, Sudan | 2013 floods |  |
| Liu et al. | Hunan Province, China | 12 floods (2005-2012) | * |
| Colston et al. | Loreto, Peru | 2011-2012 La Niña flooding |  |
| Balikuddembe et al. | Ethiopia, Kenya, Somalia, Sudan & Tanzania | 205 floods (1990-2019) | * |
| Bhunia & Ghosh | West Bengal, India | Cyclone Aila |  |
| Fredrik et al. | India | Cyclone Thane |  |
| Jones et al. | Guadalcanal, Solomon Islands | 2014 Tropical Depression |  |
| Wu et al. | Bangladesh | Heatwaves (1983-2009) | * |
| Li et al. | Guangdong Province, China | 20 tropical cyclones (2013-2018) | * |
| Tohme et al. | Haiti | 2010 Haiti earthquake |  |
| Ahmad et al. | Pakistan | October 2015 Hindu Kush earthquake |  |
| Thapa et al. | Nepal | April 2015 Nepal earthquake |  |
| Khanal | Eastern, Central and Western Regions of Nepal | 2015 Gorkha earthquake |  |
| Colón-Lopez et al. | Puerto Rico, United States | Hurricane Maria |  |
| Fernandes et al. | Mozambique | Cyclone Idai |  |
| Nagata et al. | 22 sub-Saharan African countries | Drought (2011-2019) | * |

***** Studies considered multiple countries and/or disaster events.
