# Supplementary figures and images for: Effects of hydro-meteorological and geological disasters on vaccine-preventable disease outbreaks and routine immunisation amongst children: A scoping review
Source: PLOS Glob Public Health. 2026 Jun 12;6(6):e0005712. doi: 10.1371/journal.pgph.0005712 (PMC13262859; doi:10.1371/journal.pgph.0005712)

**
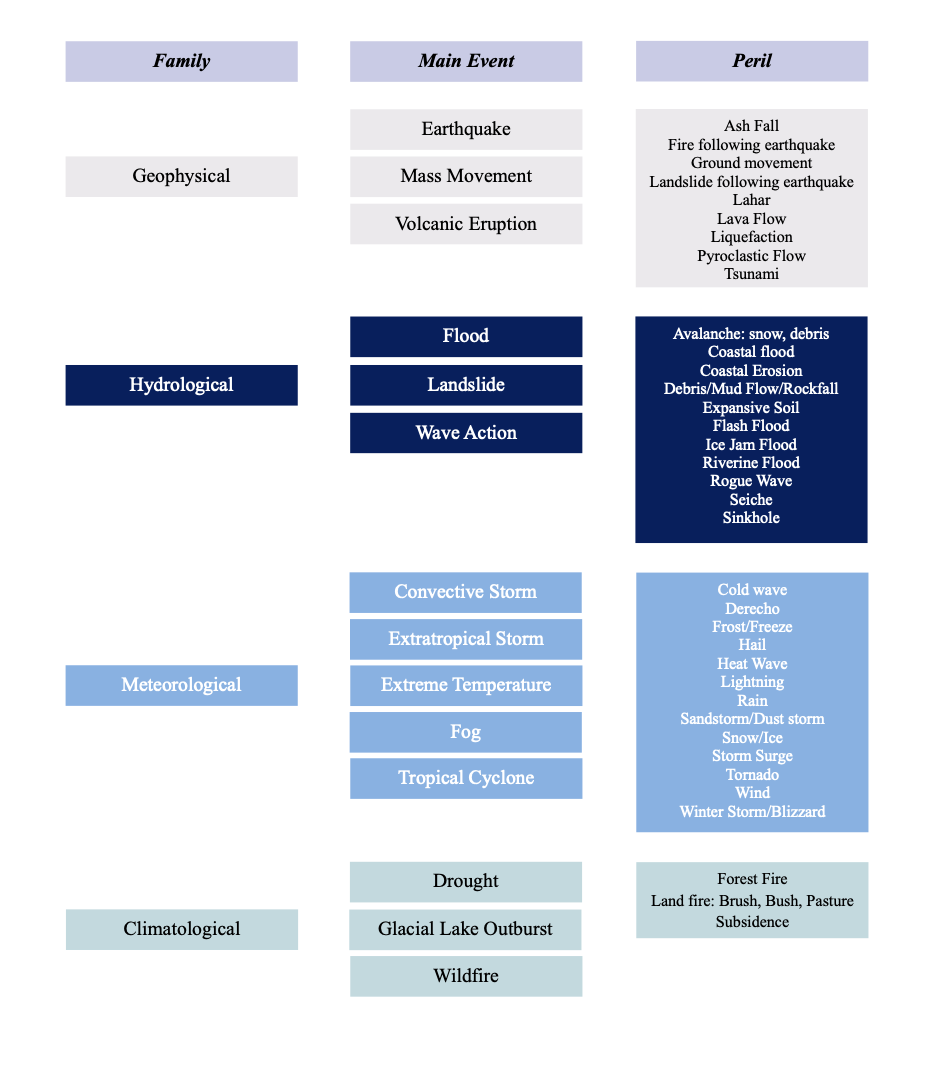
**

**Figure S1.** *IRDR disaster classification.*

Supplement: S1 Fig — (DOCX) [file pgph.0005712.s004.docx]
