# Supplementary material for: Effects of hydro-meteorological and geological disasters on vaccine-preventable disease outbreaks and routine immunisation amongst children: A scoping review
Source: PLOS Glob Public Health. 2026 Jun 12;6(6):e0005712. doi: 10.1371/journal.pgph.0005712 (PMC13262859; doi:10.1371/journal.pgph.0005712)
Supplement: Attachment — Submitted filename: Response (Edited).docx [file pgph.0005712.s005.docx]

**Reviewers comments and answers**

**Reviewer #1:**

Overall, this manuscript presents a well-structured and comprehensive scoping review on the effects of disasters on VPD outbreaks and routine immunization on children. The manuscript is well written and methodologically sound. The objectives are clear and the results are well presented. Very minor suggestions:

- Consider expanding the discussion of limitations due to language restrictions and exclusion of unpublished studies.

R: Thank you. We have now clarified that in the discussion section “Despite the strengths of our study, it is not without its limitations. Firstly, despite excluding very few studies based on language, our search terms were conducted in English, which may have excluded the literature not indexed in English.” and “Lastly, the criteria for the studies to be published may have meant the research largely came from high-income nations, losing generalisability.”

- While the conclusion infers to the implication of the finding, consider expanding on policy implications

R: We have expanded on the policy implications upon recommendation in the last paragraph of the discussion: “Evidence indicating vaccination is more critical now in light of climate change than ever before comes at a time when vaccine hesitancy is a major threat to global health (60)(61). The prevalence of VPDs is expected to increase yet American states are removing their vaccines mandates in schools and places of employment (60)(62). Some States in the U.S, for example, have abolished vaccine mandates, inspiring deep concern from public health experts especially taking into consideration the frequency of hurricanes in the state (62). This review supports vaccine mandates to ensure proper coverage and avoid unnecessary suffering, especially when it comes to disaster-prone areas.”

**Reviewer #2:**

Thank you for the opportunity to review this manuscript, which reports on a scoping review of the impacts of natural disasters on vaccine-preventable disease (VPD) outbreaks and routine immunisation of children. The paper title should specify the types of disasters that constitute the focus of the study. By using the blanket term “disasters” in the title and throughout the paper, the authors imply a broad range of disaster types. However, the study specifically focuses on a subset of natural disasters. That itself is not an issue. However, an estimated 1.1 billion people live within 5 km of active armed conflict – 14% of the global population (see ACLED: [https://acleddata.com/platform/conflict-exposure-calculator](https://url.uk.m.mimecastprotect.com/s/N-EXCyXjgiGp7gnfQi6uxju1k?domain=acleddata.com)). As it is currently presented, the title and much of the wording effectively dismisses this 14%, who also face VPD outbreaks and barriers to immunisation related to armed conflict. That figure does not include people living >50 km from active conflict, or people displaced by conflict. The authors should provide justification for why they focus specifically on certain natural disasters, and the types of disasters should be specified consistently throughout. Alternatively, the terminology should be clarified early in the introduction to avoid any confusion or mass dismissal of a significant proportion of the global population.

R: Thank you for the feedback. We have changed the title to specify that the disasters discussed are hydro-meteorological and geological in nature. We also specified that this is the type of disaster considered in the review in the final paragraph of the introduction: “We conducted a global scoping review aimed at compiling the literature available concerning the effect of hydro-meteorological and geological disasters on routine immunisation and VPD outbreaks amongst children, or those under eighteen.”

We have justified in our introduction why we have limited our review to discussion of hydro-meteorological and geological: “Given the increasing risk of hydrometeorological and geological disasters, and the urgent need for climate adaptation, particularly in regions that already bear a disproportionate burden of infectious diseases, it is imperative to understand how these disasters affect childhood vaccination and the occurrence of VPD outbreaks in order to better protect children’s health.”

Furthermore, the paper would be more effective it acknowledged the impacts of conflict and displacement in the introduction, and then focused some the discussion to consider how the study findings compare with disasters involving armed conflict and/or forced migration. There is a significant literature on this topic, which could bring an important perspective to the findings of this study.

R: We expanded our consideration of conflict and displacement in the discussion portions of this review. In the introduction, we justified why we limited the disasters to those caused by hydro-meteorological and geological events (as seen above).

In the discussion, we acknowledged the challenges to vaccination and vaccine-preventable disease prevention in conflict settings despite this disaster not being the focus of our review: “While not considered in-depth in the studies of our review, coinciding or recent conflict can further amplify the effect of disaster on VPD spread and routine immunisation.”

Additionally, we made note of how a great portion of the world’s unvaccinated children live in conflict settings: “Notably, the United Nations Children’s Fund (UNICEF) reports that close to 40% of unvaccinated or under-vaccinated children globally reside in countries experiencing conflict (51).”

It is interesting that the paper only mentions conflict a single time in the discussion, worded in a way that suggests conflict may be a moderating or exacerbating factor in reducing immunisation coverage and contributing to outbreaks of vaccine preventable diseases. Similarly, forced migration is hardly addressed, and the well-described intersections between climate change, armed conflict and forced migration are entirely absent. Both conflict and forced migration are well-studied and widely recognised contributors to: a) reduced immunisation coverage, as well as the b) incidence, c) prevalence, and d) course of VPD outbreaks. Indeed, even some of the studies included in this review draw attention to this. The almost dismissive approach to armed conflict and forced displacement seriously undermines this manuscript. It may be helpful for the authors to review the yearly UNHCR reports on Global Trends in Forced Displacement, and some of the review papers in the literature on conflict and health.

R: We drew attention to the intersection between climate change and conflict in the discussion of Cyclones Idai and Kenneth, “In Mozambique, Cyclones Idai and Kenneth came at a time of civil unrest, specifically armed conflict in regions such as Sofala (50). These combined tragedies increased population displacement and forced internal and external migration and the interruption of healthcare services (50). Conflicts can additionally leave a population quite vulnerable to disaster-related VPD outbreaks, as the healthcare system may be weakened, the population under-vaccinated, and the government unprepared to control the spread.” Further attention may be beyond the scope of this review.

Lastly, the paper would be strengthened if it clarified how it contributes to the existing literature. Much of the findings mirror those seen in other kinds of disaster settings. What is the added value of this study?

R: We have justified the need for this study in the introduction: “Given the increasing risk of hydrometeorological and geological disasters, and the urgent need for climate adaptation, particularly in regions that already bear a disproportionate burden of infectious diseases, it is imperative to understand how these disasters affect childhood vaccination and the occurrence of VPD outbreaks in order to better protect children’s health.”

We have further pointed to its need in the discussion: “This is critical research, as disasters invite conditions ideal for VPD outbreaks, especially in low-resource settings and amongst socially vulnerable populations and communities. These events can further disrupt routine immunisation, particularly when health systems are fragile.”

Some additional comments:

1. Please include line numbers to facilitate clear and precise feedback.

Methods
2. Please provide a description of the search terms used across the databases (i.e. variants of…) in the manuscript text. It is fine to provide a complete search strategy in the supplementary information. I’m unfortunately not able to access the supplementary information to review the information provided. Most readers are also not likely to consult the supplementary information.

R: We have now included in the “Data sources and search” section how these search terms were organized, “Search terms fell under four concepts: vaccines (names for WHO-recommended vaccines), vaccine-preventable diseases (the diseases covered by WHO-recommended vaccines), disasters (those satisfying EM-DAT criteria), and children (synonyms for different child age groups).”

3. Page 6, paragraph 2: Were tables 2 & 3 created and then used to analyse the findings?

R: We have now clarified that in the methods section: “After full-text screening, a single reviewer (IB) extracted the following data in a pre-defined extraction form containing: author, year published, title, study aims, study design, study setting, type of disaster, date(s) of disaster, vaccine or VPD of interest, populations affected and unaffected, outcome for affected and unaffected populations, main results and conclusions.

Following data extraction, the findings were summarised in Tables by IRDR-classified disaster type (See Figure S1), and using a narrative synthesis.”

4. Please provide further information about the method of analysis.

R: We have now provided a better description on the structure of the narrative synthesis “Following data extraction, the findings were summarised in tables by IRDR-classified disaster type (See Figure S1) and in a narrative synthesis. The narrative synthesis followed JBI guidelines and was organised to match study objectives, which included two topics on the overall effects of disaster-related disruption on (i) childhood immunisation programmes and on (ii) VPD outbreaks by regions of study, publication years, disasters in question, vaccine types, and diseases covered; and three topics aimed to go in depth on the effects of social and demographic factors, key disaster/health events relationships (i.e., impact of floods on WASH-related and vector-borne VPDs), and common disaster-induced obstacles to routine childhood immunisation.”

Results
5. Page 17 paragraph 2 is confusing. How many were included after screening? Was a subset of studies from 1991-2024 included? And were previous studies rejected? If so, why? If a subset were analysed, the justification should be provided and the decision-making process for this provided in the methods.

R: We have clarified what we meant by the years given, “The studies selected were all published between 1991 and 2024 (See Tables 2 & 3). 12 studies were published between 2010–2019, studies from the 1990s and 2000s were rather sparse.”

6. Page 18 – the first section may be more appropriately titled social determinants or social and demographic factors, as it also mentions poverty and deprivation

R: This section was renamed to “Investigation of outbreaks by social and demographic factors.”

7. Page 18, there is a short paragraph on nutritional status – this should be expanded with reporting on findings.

R: We limited this discussion to a short paragraph, as nutritional status was solely mentioned in this studies as a vulnerability factor.

8. Additionally, the two sentences about malnutrition impacts on immune system and VPD outcomes belong in discussion and should be supported with citations. Malnutrition is a complex multiorgan system disease, including immune functioning among other organ systems. There is a fair bit of literature on humanitarian crises and immunisation. Studies on VPD and/or immunisation in humanitarian settings typically look at links between immunisation status and nutrition outcomes, and some studies evaluate the impacts of combined nutrition-immunisation programmes. There are even randomised controlled trials looking at vaccine immunogenic response in undernourished children. These would all be worthwhile topics to explore in the discussion.

R: The conversation concerning malnutrition has now been moved completely to the discussion portion of our review: “Despite minimal attention from the studies included in our review, malnutrition can further be exacerbated in disaster time and affect children’s susceptibility to infection (48). Poor nutritional status and infection can be cyclical in nature - malnutrition invites infection and infection may bring about malnutrition (49) ...”

Discussion
Please see my comments above. This section should be overhauled to address the elephant in the room – all the other kinds of disasters, some of which are sadly far more prevalent and chronic, compared with the subset of natural disasters that this study focused on. There is a literature on conflict, displacement and impacts on immunisation and outbreaks, which albeit still limited, has a number of useful studies and provides a useful comparator to the findings of the studies included in this review.

R: Thank you for the comment. We agree with the point and we have now better discussed this in the discussion section “While not considered in-depth in the studies of our review, coinciding or recent conflict can further amplify the effect of disaster on VPD spread and routine immunisation. In Mozambique, Cyclones Idai and Kenneth came at a time of civil unrest, specifically armed conflict in regions such as Sofala (50) ... Notably, the United Nations Children’s Fund (UNICEF) reports that close to 40% of unvaccinated or under-vaccinated children globally reside in countries experiencing conflict (51).” At the same time, we kept the conversation to one paragraph to avoid going beyond the scope of our review.

**Reviewer #3:**

Thank you for giving me the opportunity to review this paper. With the effects of climate change and increasing weather events leading to disasters, this paper is timely and relevant. I found it to be well written and structured.

I have the following minor comments:

Materials and methods:

- Definitions: The authors should consider breaking down, especially the 2nd sentence discussing the definitions, into smaller sentences as it’s a bit difficult to read and digest.

R: We have reduced the sentence to our best ability to read as follows “In summary, we looked at the effect of exposure to a simplified classification of EM-DAT recognised and Integrated Research on Disaster Risk (IRDR)-classified disaster events or perils on the coverage and uptake of WHO recommended childhood vaccines or the cases, deaths, and measures of association for the 23 corresponding VPDs amongst UNCRC-defined children globally (See Table 1 for PICO strategy and Figure S1 for EM-DAT disaster definition).”

- Please define IRDR in this section as this is the first time it appears in the paper.

R: This has been amended (see above).

Supplementary/supporting information: The authors should consider revising the page numbers found in the PRISMA checklists in both supplementary files as the page numbers do not always align with the checklist items.

R: This has been amended, thank you.
