# Supplementary material for: Effects of hydro-meteorological and geological disasters on vaccine-preventable disease outbreaks and routine immunisation amongst children: A scoping review
Source: PLOS Glob Public Health. 2026 Jun 12;6(6):e0005712. doi: 10.1371/journal.pgph.0005712 (PMC13262859; doi:10.1371/journal.pgph.0005712)
Supplement: Peer review history

# Peer review: Decision Letter 0

**DOI:** 10.1371/journal.pgph.0005712.r001

**Authors:** Michele Nguyen

15 Oct 2025

PGPH-D-25-01982

Effects of disasters on vaccine-preventable disease outbreaks and routine immunisation amongst children: a scoping review

PLOS Global Public Health

Dear Dr. Pescarini,

Thank you for submitting your manuscript to PLOS Global Public Health. After careful consideration, we feel that it has merit but does not fully meet PLOS Global Public Health’s publication criteria as it currently stands. Therefore, we invite you to submit a revised version of the manuscript that addresses the points raised during the review process.

As mentioned by a reviewer, there is a need to be more specific about the type of disasters covered in the title and the manuscript, and justify the choice to focus on these instead of other types of disasters. It is also advisable to highlight the impact of other types of disasters and forced migration on vaccine-preventable disease outbreaks especially in the discussion.

Please submit your revised manuscript by Nov 29 2025 11:59PM. If you will need more time than this to complete your revisions, please reply to this message or contact the journal office at globalpubhealth@plos.org. When you're ready to submit your revision, log on to https://www.editorialmanager.com/pgph/ and select the 'Submissions Needing Revision' folder to locate your manuscript file.

Please include the following items when submitting your revised manuscript:

- A rebuttal letter that responds to each point raised by the editor and reviewer(s). You should upload this letter as a separate file labeled 'Response to Reviewers'.
- A marked-up copy of your manuscript that highlights changes made to the original version. You should upload this as a separate file labeled 'Revised Manuscript with Track Changes'.
- An unmarked version of your revised paper without tracked changes. You should upload this as a separate file labeled 'Manuscript'.

Guidelines for resubmitting your figure files are available below the reviewer comments at the end of this letter.

We look forward to receiving your revised manuscript.

Kind regards,

Michele Nguyen

Academic Editor

PLOS Global Public Health

Journal Requirements:

If the reviewer comments include a recommendation to cite specific previously published works, please review and evaluate these publications to determine whether they are relevant and should be cited. There is no requirement to cite these works unless the editor has indicated otherwise.

Additional Editor Comments (if provided):

[Note: HTML markup is below. Please do not edit.]

Reviewers' comments:

Reviewer's Responses to Questions

**Comments to the Author**

1. Does this manuscript meet PLOS Global Public Health’s publication criteria? Is the manuscript technically sound, and do the data support the conclusions? The manuscript must describe methodologically and ethically rigorous research with conclusions that are appropriately drawn based on the data presented.

Reviewer #1: Yes

Reviewer #2: Partly

Reviewer #3: Yes

**********

2. Has the statistical analysis been performed appropriately and rigorously?

Reviewer #1: Yes

Reviewer #2: N/A

Reviewer #3: Yes

**********

3. Have the authors made all data underlying the findings in their manuscript fully available (please refer to the Data Availability Statement at the start of the manuscript PDF file)?

The PLOS Data policy requires authors to make all data underlying the findings described in their manuscript fully available without restriction, with rare exception. The data should be provided as part of the manuscript or its supporting information, or deposited to a public repository. For example, in addition to summary statistics, the data points behind means, medians and variance measures should be available. If there are restrictions on publicly sharing data—e.g. participant privacy or use of data from a third party—those must be specified.

Reviewer #1: No

Reviewer #2: Yes

Reviewer #3: Yes

**********

4. Is the manuscript presented in an intelligible fashion and written in standard English?

PLOS Global Public Health does not copyedit accepted manuscripts, so the language in submitted articles must be clear, correct, and unambiguous. Any typographical or grammatical errors should be corrected at revision, so please note any specific errors here.

Reviewer #1: Yes

Reviewer #2: Yes

Reviewer #3: Yes

**********

5. Review Comments to the Author

Please use the space provided to explain your answers to the questions above. You may also include additional comments for the author, including concerns about dual publication, research ethics, or publication ethics. (Please upload your review as an attachment if it exceeds 20,000 characters)

Reviewer #1: Overall, this manuscript presents a well-structured and comprehensive scoping review on the effects of disasters on VPD outbreaks and routine immunization on children. The manuscript is well written and methodologically sound. The objectives are clear and the results are well presented.

Very minor suggestions:

- Consider expanding the discussion of limitations due to language restrictions and exclusion of unpublished studies.

- While the conclusion infers to the implication of the finding, consider expanding on policy implications

Reviewer #2: Thank you for the opportunity to review this manuscript, which reports on a scoping review of the impacts of natural disasters on vaccine-preventable disease (VPD) outbreaks and routine immunisation of children.

The paper title should specify the types of disasters that constitute the focus of the study. By using the blanket term “disasters” in the title and throughout the paper, the authors imply a broad range of disaster types. However, the study specifically focuses on a subset of natural disasters. That itself is not an issue. However, an estimated 1.1 billion people live within 5 km of active armed conflict – 14% of the global population (see ACLED: https://acleddata.com/platform/conflict-exposure-calculator). As it is currently presented, the title and much of the wording effectively dismisses this 14%, who also face VPD outbreaks and barriers to immunisation related to armed conflict. That figure does not include people living >50 km from active conflict, or people displaced by conflict. The authors should provide justification for why they focus specifically on certain natural disasters, and the types of disasters should be specified consistently throughout. Alternatively, the terminology should be clarified early in the introduction to avoid any confusion or mass dismissal of a significant proportion of the global population.

Furthermore, the paper would be more effective it acknowledged the impacts of conflict and displacement in the introduction, and then focused some the discussion to consider how the study findings compare with disasters involving armed conflict and/or forced migration. There is a significant literature on this topic, which could bring an important perspective to the findings of this study.

it is interesting that the paper only mentions conflict a single time in the discussion, worded in a way that suggests conflict may be a moderating or exacerbating factor in reducing immunisation coverage and contributing to outbreaks of vaccine preventable diseases. Similarly, forced migration is hardly addressed, and the well-described intersections between climate change, armed conflict and forced migration are entirely absent. Both conflict and forced migration are well-studied and widely recognised contributors to: a) reduced immunisation coverage, as well as the b) incidence, c) prevalence, and d) course of VPD outbreaks. Indeed, even some of the studies included in this review draw attention to this. The almost dismissive approach to armed conflict and forced displacement seriously undermines this manuscript. It may be helpful for the authors to review the yearly UNHCR reports on Global Trends in Forced Displacement, and some of the review papers in the literature on conflict and health.

Lastly, the paper would be strengthened if it clarified how it contributes to the existing literature. Much of the findings mirror those seen in other kinds of disaster settings. What is the added value of this study?

Some additional comments:

1. Please include line numbers to facilitate clear and precise feedback.

Methods

2. Please provide a description of the search terms used across the databases (i.e. variants of…) in the manuscript text. It is fine to provide a complete search strategy in the supplementary information. I’m unfortunately not able to access the supplementary information to review the information provided. Most readers are also not likely to consult the supplementary information.

3. Page 6, paragraph 2: Were tables 2 & 3 created and then used to analyse the findings?

4. Please provide further information about the method of analysis.

Results

5. Page 17 paragraph 2 is confusing. How many were included after screening? Was a subset of studies from 1991-2024 included? And were previous studies rejected? If so, why? If a subset were analysed, the justification should be provided and the decision-making process for this provided in the methods.

6. Page 18 – the first section may be more appropriately titled social determinants or social and demographic factors, as it also mentions poverty and deprivation

7. Page 18, there is a short paragraph on nutritional status – this should be expanded with reporting on findings.

8. Additionally, the two sentences about malnutrition impacts on immune system and VPD outcomes belong in discussion and should be supported with citations. Malnutrition is a complex multiorgan system disease, including immune functioning among other organ systems. There is a fair bit of literature on humanitarian crises and immunisation. Studies on VPD and/or immunisation in humanitarian settings typically look at links between immunisation status and nutrition outcomes, and some studies evaluate the impacts of combined nutrition-immunisation programmes. There are even randomised controlled trials looking at vaccine immunogenic response in undernourished children. These would all be worthwhile topics to explore in the discussion.

Discussion

Please see my comments above. This section should be overhauled to address the elephant in the room – all the other kinds of disasters, some of which are sadly far more prevalent and chronic, compared with the subset of natural disasters that this study focused on. There is a literature on conflict, displacement and impacts on immunisation and outbreaks, which albeit still limited, has a number of useful studies and provides a useful comparator to the findings of the studies included in this review.

Reviewer #3: Thank you for giving me the opportunity to review this paper. With the effects of climate change and increasing weather events leading to disasters, this paper is timely and relevant. I found it to be well written and structured.

I have the following minor comments:

Materials and methods

- Definitions: The authors should consider breaking down, especially the 2nd sentence discussing the definitions, into smaller sentences as it’s a bit difficult to read and digest.

- Please define IRDR in this section as this is the first time it appears in the paper.

Supplementary/supporting information

- The authors should consider revising the page numbers found in the PRISMA checklists in both supplementary files as the page numbers do not always align with the checklist items.

**********

6. PLOS authors have the option to publish the peer review history of their article (what does this mean?). If published, this will include your full peer review and any attached files.

**Do you want your identity to be public for this peer review?** If you choose “no”, your identity will remain anonymous but your review may still be made public.

For information about this choice, including consent withdrawal, please see our Privacy Policy.

Reviewer #1: No

Reviewer #2: No

Reviewer #3: **Yes:** Grace Mambula

**********

[NOTE: If reviewer comments were submitted as an attachment file, they will be attached to this email and accessible via the submission site. Please log into your account, locate the manuscript record, and check for the action link "View Attachments". If this link does not appear, there are no attachment files.]

-->Figure Resubmissions:

While revising your submission, we strongly recommend that you use PLOS’s NAAS tool (https://ngplosjournals.pagemajik.ai/artanalysis) to test your figure files. NAAS can convert your figure files to the TIFF file type and meet basic requirements (such as print size, resolution), or provide you with a report on issues that do not meet our requirements and that NAAS cannot fix.

After uploading your figures to PLOS’s NAAS tool - https://ngplosjournals.pagemajik.ai/artanalysis, NAAS will process the files provided and display the results in the "Uploaded Files" section of the page as the processing is complete. If the uploaded figures meet our requirements (or NAAS is able to fix the files to meet our requirements), the figure will be marked as "fixed" above. If NAAS is unable to fix the files, a red "failed" label will appear above. When NAAS has confirmed that the figure files meet our requirements, please download the file via the download option, and include these NAAS processed figure files when submitting your revised manuscript.

# Author comment: Author response to Decision Letter 1

**DOI:** 10.1371/journal.pgph.0005712.r002

9 Dec 2025

Attachment Submitted filename: Response (Edited).docx

# Peer review: Decision Letter 1

**DOI:** 10.1371/journal.pgph.0005712.r003

**Authors:** Inayat Ali

13 Jan 2026

PGPH-D-25-01982R1

Effects of hydro-meteorological and geological disasters on vaccine-preventable disease outbreaks and routine immunisation amongst children: a scoping review

PLOS Global Public Health

Dear Dr. Pescarini,

Thank you for submitting your manuscript to PLOS Global Public Health. After careful consideration, we feel that it has merit but does not fully meet PLOS Global Public Health’s publication criteria as it currently stands. Therefore, we invite you to submit a revised version of the manuscript that addresses the points raised during the review process.

Please submit your revised manuscript by Feb 27 2026 11:59PM. If you will need more time than this to complete your revisions, please reply to this message or contact the journal office at globalpubhealth@plos.org. When you're ready to submit your revision, log on to https://www.editorialmanager.com/pgph/ and select the 'Submissions Needing Revision' folder to locate your manuscript file.

Please include the following items when submitting your revised manuscript:

- A letter that responds to each point raised by the editor and reviewer(s). You should upload this letter as a separate file labeled 'Response to Reviewers'.
- A marked-up copy of your manuscript that highlights changes made to the original version. You should upload this as a separate file labeled 'Revised Manuscript with Track Changes'.
- An unmarked version of your revised paper without tracked changes. You should upload this as a separate file labeled 'Manuscript'.

Guidelines for resubmitting your figure files are available below the reviewer comments at the end of this letter.

We look forward to receiving your revised manuscript.

Kind regards,

Inayat Ali, B.A., M.Sc., M.Phil., PhD

Academic Editor

PLOS Global Public Health

Journal Requirements:

1. If the reviewer comments include a recommendation to cite specific previously published works, please review and evaluate these publications to determine whether they are relevant and should be cited. There is no requirement to cite these works unless the editor has indicated otherwise.

Additional Editor Comments (if provided):

[Note: HTML markup is below. Please do not edit.]

Reviewers' comments:

Reviewer's Responses to Questions

**Comments to the Author**

1. If the authors have adequately addressed your comments raised in a previous round of review and you feel that this manuscript is now acceptable for publication, you may indicate that here to bypass the “Comments to the Author” section, enter your conflict of interest statement in the “Confidential to Editor” section, and submit your "Accept" recommendation.

Reviewer #2: (No Response)

Reviewer #3: All comments have been addressed

**********

2. Does this manuscript meet PLOS Global Public Health’s publication criteria? Is the manuscript technically sound, and do the data support the conclusions? The manuscript must describe methodologically and ethically rigorous research with conclusions that are appropriately drawn based on the data presented.

Reviewer #2: Partly

Reviewer #3: Yes

**********

3. Has the statistical analysis been performed appropriately and rigorously?

Reviewer #2: N/A

Reviewer #3: Yes

**********

4. Have the authors made all data underlying the findings in their manuscript fully available (please refer to the Data Availability Statement at the start of the manuscript PDF file)?

The PLOS Data policy requires authors to make all data underlying the findings described in their manuscript fully available without restriction, with rare exception. The data should be provided as part of the manuscript or its supporting information, or deposited to a public repository. For example, in addition to summary statistics, the data points behind means, medians and variance measures should be available. If there are restrictions on publicly sharing data—e.g. participant privacy or use of data from a third party—those must be specified.

Reviewer #2: Yes

Reviewer #3: Yes

**********

5. Is the manuscript presented in an intelligible fashion and written in standard English?

PLOS Global Public Health does not copyedit accepted manuscripts, so the language in submitted articles must be clear, correct, and unambiguous. Any typographical or grammatical errors should be corrected at revision, so please note any specific errors here.

Reviewer #2: Yes

Reviewer #3: Yes

**********

6. Review Comments to the Author

Please use the space provided to explain your answers to the questions above. You may also include additional comments for the author, including concerns about dual publication, research ethics, or publication ethics. (Please upload your review as an attachment if it exceeds 20,000 characters)

Reviewer #2: Thank you for the opportunity to review the revised manuscript. It is improved now, especially in the framing of the focus. There are still a few major problems.

Results:

The results section should present the findings of the studies. Relevant discussion and conclusions from the included papers may be explored in the discussion section. At present, quite a bit of the results presents factors that authors for the included studies speculated on or discussed – not their study findings. For example, “After the Kashmir earthquake, Karmakar et al. noted high rotavirus infections amongst infants (16). The authors suggested that given that this age group may not have directly consumed contaminated water (a source of the outbreak), speculating that contraction largely resulted from contact with a care provider (16)”. Or, “In their 2006 study of diarrhoea amongst Bangladeshi children during flooding, Schwartz et al. suggested refugees could be an especially susceptible population when it came to falling ill during a cholera outbreak (22). The authors pointed to the 1994 diarrhoeal epidemic in Goma, Zaire amongst Rwandan refugees, speculated to have been possible because of contaminated water in addition to inadequate sanitation and hygiene services and education (42).”

At one point, the authors write a conclusion in the results section, “With flooding and elevated water levels in the area, it is likely that faecal matter entered water streams.”

This occurs through the results section. The section should be revised to only present study findings, and move the discussion to the discussion section.

Discussion:

The authors don’t critically examine the findings of their review. Instead, the discussion section focuses on summarising and making conclusions and recommendations. This is perhaps in part because some of the discussion is presented in the results section. Both sections should be reworked to correct this.

As I noted in my previous feedback, similar trends in reduced immunisation coverage and increased vaccine-preventable disease outbreaks are also found in other kinds of disasters, including forced displacement (internally displaced as well as refugees), and in conflict-affected settings (peri- and post-conflict). This is well-documented by reviews in different populations, and these reviews often include further contextual information which are relevant to the potential mechanisms and factors at play. The findings from this scoping review should, at the very least, be compared with the findings of the existing reviews on vaccination coverage and VPD outbreaks in other kinds of crises.

Please note the current wording on page 21 first paragraph implies that conflict is an amplifier of the effects of hydro-meteorological and geological disasters on vaccine-preventable diseases. The authors provide no evidence to support this statement. There is a rather large body of evidence for how conflict and displacement affect vaccination coverage and VPD outbreaks in contexts where there is not a proximate hydro-meteorological and/or geological disaster. If the authors wish to propose conflict as a mediator, they should provide strong evidence to support this statement.

Minor comment:

Table 1 subtext is incorrect. BCG (TB vaccination) is not routinely recommended by the WHO for all contexts. It is recommended for all children countries with high disease burden, and for high-risk populations in low burden contexts. The text should be corrected to reflect this.

Reviewer #3: (No Response)

**********

7. PLOS authors have the option to publish the peer review history of their article (what does this mean?). If published, this will include your full peer review and any attached files.

**Do you want your identity to be public for this peer review?** If you choose “no”, your identity will remain anonymous but your review may still be made public.

For information about this choice, including consent withdrawal, please see our Privacy Policy.

Reviewer #2: No

Reviewer #3: **Yes:** Grace Mambula

**********

[NOTE: If reviewer comments were submitted as an attachment file, they will be attached to this email and accessible via the submission site. Please log into your account, locate the manuscript record, and check for the action link "View Attachments". If this link does not appear, there are no attachment files.]

-->Figure Resubmissions:

While revising your submission, we strongly recommend that you use PLOS’s NAAS tool (https://ngplosjournals.pagemajik.ai/artanalysis) to test your figure files. NAAS can convert your figure files to the TIFF file type and meet basic requirements (such as print size, resolution), or provide you with a report on issues that do not meet our requirements and that NAAS cannot fix.

After uploading your figures to PLOS’s NAAS tool - https://ngplosjournals.pagemajik.ai/artanalysis, NAAS will process the files provided and display the results in the "Uploaded Files" section of the page as the processing is complete. If the uploaded figures meet our requirements (or NAAS is able to fix the files to meet our requirements), the figure will be marked as "fixed" above. If NAAS is unable to fix the files, a red "failed" label will appear above. When NAAS has confirmed that the figure files meet our requirements, please download the file via the download option, and include these NAAS processed figure files when submitting your revised manuscript.

# Author comment: Author response to Decision Letter 2

**DOI:** 10.1371/journal.pgph.0005712.r004

16 Feb 2026

Attachment Submitted filename: Responses to the Reviewers.docx

# Peer review: Decision Letter 2

**DOI:** 10.1371/journal.pgph.0005712.r005

**Authors:** Inayat Ali

18 May 2026

Effects of hydro-meteorological and geological disasters on vaccine-preventable disease outbreaks and routine immunisation amongst children: a scoping review

PGPH-D-25-01982R2

Dear Miss Pescarini,

We are pleased to inform you that your manuscript 'Effects of hydro-meteorological and geological disasters on vaccine-preventable disease outbreaks and routine immunisation amongst children: a scoping review' has been provisionally accepted for publication in PLOS Global Public Health.

Before your manuscript can be formally accepted you will need to complete some formatting changes, which you will receive in a follow up email. A member of our team will be in touch with a set of requests.

Please note that your manuscript will not be scheduled for publication until you have made the required changes, so a swift response is appreciated.

IMPORTANT: The editorial review process is now complete. PLOS will only permit corrections to spelling, formatting or significant scientific errors from this point onwards. Requests for major changes, or any which affect the scientific understanding of your work, will cause delays to the publication date of your manuscript.

If your institution or institutions have a press office, please notify them about your upcoming paper to help maximize its impact. If they'll be preparing press materials, please inform our press team as soon as possible -- no later than 48 hours after receiving the formal acceptance. Your manuscript will remain under strict press embargo until 2 pm Eastern Time on the date of publication. For more information, please contact globalpubhealth@plos.org.

Thank you again for supporting Open Access publishing; we are looking forward to publishing your work in PLOS Global Public Health.

Best regards,

Inayat Ali, B.A., M.Sc., M.Phil., PhD

Academic Editor

PLOS Global Public Health

***********************************************************

Dear authors,

thank you for your efforts to improve your paper in light of previous comments. I am pleased to inform you that your paper has been accepted with a few minor changes to be made. The more important among them includes as highlighted by the reviewer: the authors could clarify how they addressed the heterogeneity between disaster types, geographic contexts, vaccines, and disease outcomes. Second, since disaster magnitude was not considered, it would be useful to briefly discuss how this may affect the interpretation of the findings?

Please make these changes!

Reviewer Comments (if any, and for reference):

Reviewer's Responses to Questions

**Comments to the Author**

1. If the authors have adequately addressed your comments raised in a previous round of review and you feel that this manuscript is now acceptable for publication, you may indicate that here to bypass the “Comments to the Author” section, enter your conflict of interest statement in the “Confidential to Editor” section, and submit your "Accept" recommendation.

Reviewer #4: (No Response)

Reviewer #5: (No Response)

**********

2. Does this manuscript meet PLOS Global Public Health’s publication criteria? Is the manuscript technically sound, and do the data support the conclusions? The manuscript must describe methodologically and ethically rigorous research with conclusions that are appropriately drawn based on the data presented.

Reviewer #4: Yes

Reviewer #5: Yes

**********

3. Has the statistical analysis been performed appropriately and rigorously?

Reviewer #4: Yes

Reviewer #5: Yes

**********

4. Have the authors made all data underlying the findings in their manuscript fully available (please refer to the Data Availability Statement at the start of the manuscript PDF file)?

The PLOS Data policy requires authors to make all data underlying the findings described in their manuscript fully available without restriction, with rare exception. The data should be provided as part of the manuscript or its supporting information, or deposited to a public repository. For example, in addition to summary statistics, the data points behind means, medians and variance measures should be available. If there are restrictions on publicly sharing data—e.g. participant privacy or use of data from a third party—those must be specified.

Reviewer #4: Yes

Reviewer #5: Yes

**********

5. Is the manuscript presented in an intelligible fashion and written in standard English?

PLOS Global Public Health does not copyedit accepted manuscripts, so the language in submitted articles must be clear, correct, and unambiguous. Any typographical or grammatical errors should be corrected at revision, so please note any specific errors here.

Reviewer #4: Yes

Reviewer #5: Yes

**********

6. Review Comments to the Author

Please use the space provided to explain your answers to the questions above. You may also include additional comments for the author, including concerns about dual publication, research ethics, or publication ethics. (Please upload your review as an attachment if it exceeds 20,000 characters)

Reviewer #4: Well articulated and references manuscript

Reviewer #5: The manuscript addresses an important and highly relevant public health issue: the impact of hydro-meteorological and geological disasters on childhood immunisation and vaccine-preventable disease outbreaks. The topic is particularly relevant as climate-related and geological disasters increasingly threaten the continuity of routine immunisation services, especially among children and other vulnerable populations.

The study is well suited to a scoping review design. The use of PRISMA-ScR guidelines, the search across five major databases, and the clearly defined inclusion and exclusion criteria strengthen the methodological basis of the review. The use of PICO, together with EM-DAT and IRDR disaster classifications, also helps define the scope of the study in a clear and systematic way.

A key strength of the manuscript is that it highlights an important gap in the literature. The small number of included studies, despite a broad search strategy, reinforces the need for this review and shows that the field remains underexplored. The manuscript also makes an important contribution by linking immunisation disruption to broader post-disaster conditions, including damage to health systems, cold-chain challenges, WASH conditions, displacement, and inequities affecting children, refugees, and minority groups.

I have a few suggestions for improvement. First, the authors could clarify how they addressed the heterogeneity between disaster types, geographic contexts, vaccines, and disease outcomes. Second, since disaster magnitude was not considered, it would be useful to briefly discuss how this may affect the interpretation of the findings. Third, the manuscript could more clearly distinguish between immunisation disruption, vaccine-preventable disease outbreaks, morbidity, and mortality as separate outcomes. Finally, the policy implications could be strengthened by offering more concrete recommendations for maintaining routine immunisation during and after disasters.

Overall, this is a valuable and timely review that contributes to an insufficiently studied area. I would recommend publication after minor revisions.

**********

7. PLOS authors have the option to publish the peer review history of their article (what does this mean?). If published, this will include your full peer review and any attached files.

**Do you want your identity to be public for this peer review?** If you choose “no”, your identity will remain anonymous but your review may still be made public.

For information about this choice, including consent withdrawal, please see our Privacy Policy.

Reviewer #4: No

Reviewer #5: No

**********
